# The Pathophysiological Role of Vascular Smooth Muscle Cells in Abdominal Aortic Aneurysm

**DOI:** 10.3390/cells14131009

**Published:** 2025-07-02

**Authors:** Dou Shi, Mo Zhang, Yuhan Zhang, Yang Shi, Xing Liu, Xianxian Wu, Zhiwei Yang

**Affiliations:** 1 Graduate School, Hebei North University, Zhangjiakou 075031, China; shidou2023@163.com (D.S.); 17826255356@163.com (M.Z.); 2 Institute of Laboratory Animal Science, Chinese Academy of Medical Sciences (CAMS) & Comparative Medicine Centre, Peking Union Medical Collage (PUMC), Beijing 100021, China; 15075863413@163.com (Y.Z.); sy_elisa@163.com (Y.S.); xingliu1976@163.com (X.L.)

**Keywords:** abdominal aortic aneurysm, vascular smooth muscle cells, pathophysiology, vascular biology, aortic lesion

## Abstract

Abdominal aortic aneurysm (AAA) is the most common aortic disease occurring below the renal arteries, caused by multiple etiologies. Currently, no effective drug treatment exists, and the specific pathogenesis remains unclear. Due to its insidious onset and diagnostic challenges, AAA often culminates in aortic rupture, which has a high mortality rate. During AAA development, vascular smooth muscle cells (VSMCs) undergo significant pathological alterations, including contractile dysfunction, phenotypic modulation, cellular degradation, and heightened inflammatory and oxidative stress responses. In particular, emerging evidence implicates vascular smooth muscle cell (VSMC) metabolic dysregulation and mitochondrial dysfunction as key contributors to AAA progression. In this review, we systematically summarize the current understanding of VSMC biology, including their developmental origins, structural characteristics, and functional roles in aortic wall homeostasis, along with the regulatory networks governing the VSMC phenotype and functional maintenance. This review highlights the urgent need for further investigation into the aortic wall VSMC pathophysiology to identify novel therapeutic targets for AAA. These insights may pave the way for innovative treatment strategies in aortic disease management.

## 1. Introduction

Aortic aneurysms, including thoracic (TAA) and abdominal (AAA) types, are the second most common aortic diseases after atherosclerosis. They are the ninth-leading cause of death worldwide, posing severe risks to human life and health [1]. Among these, AAA is the most prevalent type, primarily affecting men aged 65 and above [2]. AAA is characterized by a localized dilation of the infrarenal aorta, defined as an aortic diameter exceeding 3.0 cm or 50% greater than the normal aorta [3]. The primary complication of AAA is aortic rupture, a fatal event with a mortality rate reaching up to 80% [4]. The asymptomatic nature of AAA prior to rupture makes diagnosis challenging [5]. The pathogenesis of AAA is the result of multiple interacting factors, involving genetic susceptibility, environmental risk factors, and complex interactions with vascular pathologies such as atherosclerosis [6]. Established risk factors for AAA include hypertension, smoking, male gender, advanced age, obesity, family history, atherosclerosis, and the presence of other large-vessel aneurysms, and these factors are incorporated into screening criteria to varying degrees across different countries [7]. Previous studies suggested that the prevalence of AAA was lower in developing or less developed countries compared to high-income countries (HICs) [8]. However, more recent evidence indicates a marked decline in AAA incidence and mortality in some HICs, largely attributable to reduced smoking rates, widespread use of antihypertensive and cardioprotective medications, and the implementation of timely imaging-based screening and diagnosis [9]. In contrast, many low- and middle-income countries (LMICs) continue to face high levels of smoking, hypertension, harmful alcohol consumption, and other cardiovascular risk factors, along with limited access to timely imaging screening [9,10,11]. These challenges have hindered similar declines in AAA burden and, in some cases, contributed to an increased global burden of the disease [9,10,11]. Surgical repair, including endovascular and open surgery, is currently the primary treatment for AAA in China [12,13]. However, patients who are ineligible for surgery face a significant risk of aortic rupture, highlighting a critical unmet clinical need. The development of pharmacological therapies to slow AAA progression and prevent rupture could provide significant benefits, particularly for patients with smaller aneurysms or those unsuitable for surgical intervention. Moreover, such therapies could help mitigate post-repair complications, such as endoleaks, thereby improving overall patient outcomes.

**Figure 1:**
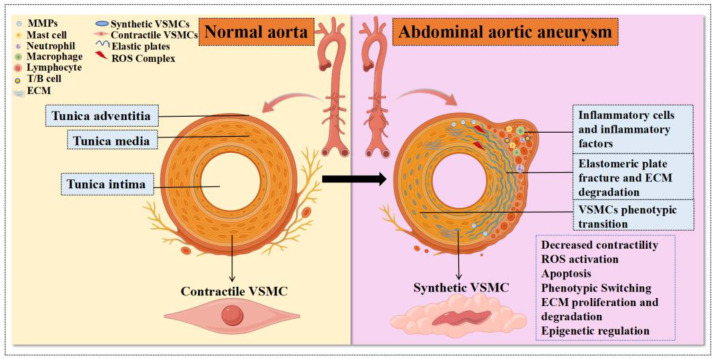
Characteristic changes in the aortic wall during the development of AAA. In the normal aorta (**left**), the vessel wall comprises three layers: the intima, media, and adventitia. Among these, VSMCs and the ECM within the medial layer are essential for maintaining the structural integrity, elasticity, and tensile strength of the aortic wall. When elastic fibers in the media are markedly reduced and VSMCs are lost or undergo apoptosis, the abdominal aorta loses mechanical support, resulting in pathological dilation and the formation of an AAA (**right**). During this pathological process, inflammatory mediators are released in a cascade, triggering and promoting the recruitment of inflammatory cells. Mitochondrial metabolic dysfunction and ROS activation within VSMCs contribute to impaired contractility. In addition, increased levels of MMPs facilitate ECM degradation, while cytokines and pro-apoptotic factors accelerate VSMC phenotypic switching, degradation, and apoptosis. Abbreviations: VSMCs: vascular smooth muscle cells; AAA: abdominal aortic aneurysm; MMPs: matrix metalloproteinases; ECM: extracellular matrix; ROS: reactive oxygen species.

Vascular smooth muscle cell (VSMC) apoptosis, extracellular matrix (ECM) degradation, chronic vascular inflammation, and oxidative stress are key pathological features of AAA, resulting in the progressive thinning of the aortic wall’s media and adventitia [14]. Vascular smooth muscle cells (VSMCs), the fundamental components of the arterial media, regulate vascular tone and blood flow to sustain the normal blood circulation and blood pressure. They play a crucial and irreplaceable role in the physiological and pathological processes of aneurysm formation [15]. ECM destruction is a critical pathophysiological mechanism of AAA, primarily mediated by matrix metalloproteinases (MMPs) [16]. MMPs are enzymes that degrade ECM components, such as collagen and elastin in the abdominal aortic wall, driving arterial wall remodeling and progressive dilation, ultimately leading to aneurysm formation [14]. Besides their association with inflammatory cell infiltration, ECM degradation and increased MMP secretion in AAA are significantly regulated by VSMCs (Figure 1).

Evidence increasingly suggests that during vascular injury and AAA formation, VSMCs undergo a phenotypic switch from a quiescent “contractile” state to a highly migratory and proliferative “synthetic” phenotype [17]. Recent genetic and epigenetic studies indicate that this phenotypic modulation, characterized by dedifferentiation and increased proliferation of VSMCs, is closely linked to ECM remodeling in the vascular wall, along with increased cell senescence and inflammation, as demonstrated in both in vitro and in vivo models [16]. Given their critical roles in vascular health and disease—such as proliferation, contraction, phenotype switching, apoptosis, ECM degradation, regeneration, and inflammation—this review focuses on VSMCs in AAA pathogenesis, exploring regulatory mechanisms, recent advances, and potential strategies for treatment and prevention.

## 2. Analysis of the Mechanism of Susceptibility to Infrarenal Aneurysms Based on VSMCs

### 2.1. Origin of VSMCs

Vasculogenesis and angiogenesis occur during early embryonic development in vertebrates, involving the coordinated actions of endothelial cells, smooth muscle cells, and pericytes [18]. The invagination of epiblastic cells through the primitive streak and the formation of the mesoderm during gastrulation drives angiogenesis [19]. Shortly thereafter, endothelial cells derived from mesodermal progenitors organize into a primitive vascular plexus, marking the onset of vascular network formation and the initiation of vascular development [18,20]. Subsequently, smooth muscle cells and pericytes are recruited to remodel the endothelium and establish a mature vascular architecture [18,21]. VSMCs originate from various mesodermal lineages, including the splanchnic mesoderm, lateral plate mesoderm, somatic or paraxial mesoderm, and neural crest [18]. In contrast, the precise origin of pericytes remains less well defined; classical studies suggest that pericytes in the aorta and coronary vasculature arise from the somitic mesoderm and epicardial mesothelium, respectively [18,22].

**Figure 2:**
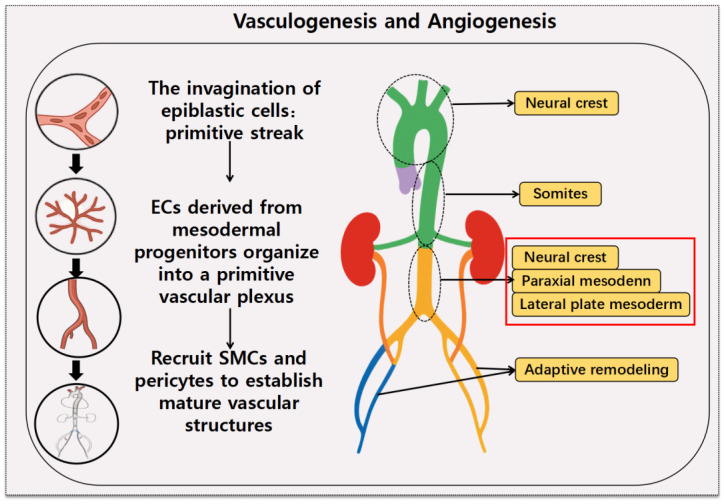
Embryonic origins of vascular smooth muscle cells in the aorta. This schematic illustrates the developmental processes of vasculogenesis and angiogenesis, highlighting the stepwise formation and maturation of the vascular system. Endothelial cells (ECs) originate from mesodermal progenitors and assemble into a primitive vascular plexus. Subsequently, smooth muscle cells (SMCs) and pericytes are recruited to stabilize and mature the vasculature. The aortic regions are color-coded, with an emphasis on the abdominal aorta (yellow), which derives its vascular smooth muscle primarily from paraxial mesoderm and lateral plate mesoderm, as indicated. This regional origin contrasts with the neural crest-derived SMCs of the ascending aorta and aortic arch, underscoring the spatial heterogeneity in vascular development. Abbreviations: SMCs: smooth muscle cells; ECs: endothelial cells.

VSMCs in different regions of the aorta originate from distinct embryonic lineages. Neural crest-derived VSMCs are predominantly localized in the ascending aorta and aortic arch. In contrast, the abdominal aorta contains few, if any, neural crest-derived VSMCs; instead, its VSMCs are primarily derived from mesodermal origins, particularly the paraxial mesoderm and lateral plate mesoderm [18,23] (Figure 2). The infrarenal region of the aorta is particularly susceptible to aneurysm formation, a phenomenon partly attributed to the embryonic mesodermal origin of VSMCs in this region. VSMCs in the infrarenal aorta are primarily derived from the mesoderm, and this embryonic lineage significantly influences their functional properties and responses to stimuli [24]. Compared to other regions, mesoderm-derived smooth muscle cells (SMCs) exhibit greater sensitivity to pro-inflammatory cytokines, such as IL-1β, which upregulates MMP expression and accelerates ECM degradation [25]. Additionally, transforming growth factor-beta (TGF-β), a cytokine known to promote cell proliferation and collagen production in neural crest-derived SMCs, lacks these stimulatory effects on mesoderm-derived SMCs [26]. This differential response further compromises ECM integrity and regenerative capacity in the infrarenal region, contributing to its predisposition to aneurysm formation [18]. Pathological studies indicate that TAAs are characterized by cystic elastic fiber degeneration, whereas AAAs are linked to severe intimal atherosclerosis, chronic inflammation, neovascularization, and medial elastic layer remodeling [27,28]. Consequently, compared to TAA, AAA exhibits more severe infiltration of inflammatory cells and structural remodeling, particularly affecting the media and adventitia of the arterial wall [27]. These intense inflammatory responses trigger the overexpression of MMPs, excessive production of reactive oxygen species (ROS), and cytokine accumulation, resulting in ECM degradation and VSMC apoptosis [29]. As a result of the distinct embryonic origins of VSMCs, the infrarenal region is inherently more susceptible to aneurysm formation compared to other regions [30].

### 2.2. Anatomy, Histological Structure, and Blood Flow Characteristics of the Infrarenal Aorta

The susceptibility of the infrarenal region to AAA is also influenced by its unique histological structure. The number of layers in the arterial media decreases progressively from the proximal thoracic aorta to the infrarenal segment, declining from 60–80 to 28–32 layers, with a concurrent reduction in elastic fiber content [30]. Compared to the suprarenal segment, the medium of the infrarenal abdominal aorta is markedly thinner, containing fewer elastic fibers, with an approximately equal ratio of elastic to collagen fibers [31]. Consequently, VSMCs in this region assume a greater role in providing structural support to the artery. However, the relatively low VSMC density in this region contributes to the increased vulnerability of the arterial wall to the loss of elasticity [31]. When VSMCs become dysfunctional—such as through phenotypic switching, where they transition to a synthetic, proliferative state, or through increased apoptosis—the structural integrity of the aortic wall is compromised [30].

The infrarenal aortic wall experiences considerable shear stress, particularly near the renal artery branches, where abrupt changes in blood flow velocity and pressure exacerbate wall stress [32]. These hemodynamic forces impose mechanical stress on VSMCs, inducing phenotype switching, which amplifies VSMC proliferation, migration, and secretion of matrix-degrading enzymes [33]. Moreover, the infrarenal aorta has a relatively limited blood supply compared to other arterial regions, especially in the adventitia [32,34]. The hypoxic microenvironment contributes to VSMCs’ dysfunction and metabolic disturbances, hindering their ability to preserve the arterial wall’s structural integrity. For instance, the abdominal aorta has relatively sparse vasa vasorum in the adventitia, and its vessel wall thickness limits oxygen diffusion from the lumen, rendering the medial layer susceptible to hypoxia, necrosis, and apoptosis [35]. These structural features collectively represent potential reasons why AAA occurs more frequently than TAA.

## 3. Cellular Architecture and Functional Regulation of Aortic VSMCs

### 3.1. Structural and Cellular Complexity of the Aortic Wall: Focus on VSMCs

The aortic wall is composed of three distinct layers: the intima (endothelial cells, basement membrane, and internal elastic lamina), the media (concentrically arranged vascular smooth muscle cells, elastic fiber layers, collagen fibers, and proteoglycan-rich extracellular matrix), and the adventitia (fibroblasts, immune cells, nerve fibers, and small vessels). In addition to these classical components, various non-traditional cell types have also been identified within the aortic wall structure. For instance, beyond the monolayer of endothelial cells and basement membrane in the intima, cells with migratory capacity—such as endothelial progenitor cells [36], vascular wall-resident stem cells [37], and Sca-1^+^/CD34^+^ cells [38]—have been detected in the subendothelial space or at the intima–media interface. Although these progenitor cells are limited in number, they may be recruited under pathological conditions such as aortic aneurysms to migrate toward the injury site, contribute to endothelial repair, reconstruct the medial structure, or secrete paracrine factors that modulate inflammation and smooth muscle cell behavior [36,37,38]. However, in the context of excessive inflammation, their regenerative functions may become impaired or exhausted, potentially exacerbating the local pathology [36,37,38]. For example, during the development of AAA, apoptosis of VSMCs, degradation of the ECM, disruption of the elastic lamina, and the release of pro-inflammatory cytokines such as IL-1β and TNF-α collectively lead to structural collapse of the aortic wall [39]. These changes hinder the migration of adventitial progenitor cells and result in inflammatory hijacking of medial VSMCs, disrupting their directional migration, interlayer communication, and structural integration.

Under physiological conditions, VSMCs play key roles in regulating vascular tone by synthesizing ECM components, such as elastin and collagen, and responding to hemodynamic stress with compliance and contractility [40]. Approximately 90% of the cells in the tunica media are VSMCs, which are concentrically arranged and spindle-shaped, characterized by well-organized actin–myosin filaments with mitochondria localized near the contractile apparatus, and defined by a high expression of canonical contractile markers—such as α-smooth muscle actin (α-SMA), smooth muscle myosin heavy chain (SM-MHC), SM22α, and calponin—as well as by their low proliferative and migratory capacity, calcium-dependent contractility, and critical role in maintaining vascular tone [15]. Notably, pericyte-like cells expressing NG2, PDGFRβ, and CD146 are present in the outer media near the vasa vasorum and within the adventitia, exhibiting reduced contractility but retaining multipotency and regenerative, migratory potential [41]. Additionally, small populations of immune cells are present in the normal aortic wall and may expand substantially under pathological conditions [42,43,44]. The subendothelial space of the intima typically lacks VSMCs; however, in disease-prone regions such as sites of atherosclerosis, VSMCs and progenitor cells from the media can migrate into the intima and differentiate into VSMC-like cells [45]. These cells exhibit a synthetic or inflammatory phenotype, marked by the expression of CD68 and osteopontin, driving MMP secretion, ECM deposition, plaque formation, and inflammatory cytokine production through enhanced interlayer migration [45]. The adventitia harbors few typical VSMCs but contains VSMC-like cells derived from myofibroblast or pericyte precursors near the media–adventitia interface, which exhibit low α-SMA expression, a high migratory capacity, and the ability to differentiate into ECM-synthesizing cells under pathological conditions [46].

Moreover, cell–cell and cell–matrix connections between different layers of the aortic wall play a critical role in coordinating SMC function and their responses to mechanical stress and inflammatory stimuli. Connexin 43 (Cx43) is the most widely expressed connexin in VSMCs, participating in intercellular electrical signal conduction, calcium wave propagation, synchronized contraction, and inflammatory signal transmission. Studies have shown that Cx43 plays a coordinating role in interlayer communication within the vascular wall, regulating SMC function and vascular tone [47]. Under hypoxic or inflammatory conditions, Cx43 expression is upregulated, modulating SMC responses to stress [48]. Meanwhile, VSMCs interact with the ECM via integrins, facilitating mechanotransduction, which affects cellular migration, proliferation, and inflammatory responses [49]. In addition, N-cadherin, a key cadherin expressed between SMCs, plays an essential role in maintaining layered structural stability, controlling cell migration, and transmitting mechanical stress signals [50,51]. The transition between these non-VSMC or progenitor-like states and VSMC-like phenotypes forms the cellular basis of VSMC phenotypic switching, which is regulated by multiple signaling pathways—this will be discussed further in the section on VSMC phenotypic modulation.

### 3.2. Regulatory Factors Influencing Aortic VSMC Contractile Function

Contractility is one of the most fundamental and defining functions of VSMCs. Loss of this contractile function can disrupt vascular tension, elevate aortic wall stress, and contribute to aneurysm formation [40]. The contractile function of VSMCs is affected by various factors, including imbalances in the matrix microenvironment, increased blood flow shear stress, and cytokine dysregulation.

#### 3.2.1. Intracellular Calcium Ion (Ca^2+^) Concentration

Intracellular calcium regulates VSMC stiffness by participating in myosin-driven contraction and calcium-dependent stiffness and adhesion processes. These processes are mediated by α-smooth muscle actin, α5β1 integrin, and integrin-dependent cell-extracellular matrix interactions [52,53]. The contractile function of VSMCs is primarily initiated by extracellular stimuli—such as angiotensin II or norepinephrine—that activate specific membrane receptors, subsequently triggering the opening of voltage-gated calcium channels (VGCCs) or inositol 1,4,5-trisphosphate (IP_3_) receptors and leading to an elevation of intracellular Ca^2+^ levels, which then bind to calmodulin to form a complex that activates myosin light chain kinase (MLCK), ultimately promoting actin–myosin interaction and cellular contraction [54].

Dysregulation of intracellular calcium homeostasis can impair VSMC contractility and has been implicated in the pathogenesis and progression of AAA. A study demonstrated that VSMCs from 21 AAA patients exhibited significantly impaired contractility compared to controls when evaluated using electric cell-substrate impedance sensing (ECIS) under ionomycin (a calcium ionophore, which can regulate calcium influx and increase intracellular calcium ion levels by directly stimulating the calcium pool) stimulation [55]. Additionally, Au and Dianaly observed that mice lacking low-density lipoprotein receptor-associated protein 1 (LRP1) exhibited aortic dilation and reduced vascular responsiveness to vasoconstrictors—including norepinephrine, U-46619 (a thromboxane receptor agonist), elevated potassium, and decreased L-type calcium release—indicating that LRP1 regulates VSMC contraction by modulating calcium signaling and thereby plays a protective role in preventing aneurysm development [56]. Previous studies have also reported a significant downregulation of CaV1.2 expression in VSMCs from AAA tissues, suggesting its potential role in VSMC dysfunction and vascular wall weakening associated with aneurysm development [52]. Meanwhile, calmodulin expression is aberrant in thoracic aortic aneurysm tissues and is associated with VSMC phenotypic switching and functional impairment [57]. Therefore, calcium is essential for maintaining VSMC contractile function, and inhibiting calcium signaling or blocking calcium influx can impair vascular smooth muscle contraction.

Moreover, studies have shown that during vascular wall injury, VSMCs undergo excitation–conversion, characterized by a downregulation of L-type calcium channels and upregulation of low-voltage activated (T-type) calcium channels and classical transient receptor potential (TRPC) channels, leading to phenotypic transformation of VSMCs [57,58]. Recent research has further demonstrated that TRPC6, a calcium-permeable channel, activates MMP9 by elevating intracellular Ca^2+^ levels, suggesting that calcium dysregulation can upregulate MMP expression and activity, thereby contributing to vascular pathology [59]. In terms of apoptosis, Yin et al. reported that the activation of Piezo1—a novel mechanosensitive ion channel—induces Ca^2+^ overload, mitochondrial damage, ROS accumulation, and VSMC apoptosis in a dose-dependent manner [60]. Therefore, disruption of calcium homeostasis can impair the contractile function of VSMCs by promoting phenotypic switching, enhancing MMP activity, and activating apoptotic signaling pathways.

#### 3.2.2. Mechanical Stimulation, Nitric Oxide (NO), and Prostacyclin (PGI_2_)

Blood flow shear stress is another key regulator of vasoconstrictive function. Tsai et al. reported that laminar flow shear stress prompts VSMCs to shift from a synthetic to a contractile phenotype by activating the release of PGI2 from endothelial cells, and the release of PPARδ and PPARα in VSMCs [61]. Similarly, Jia et al. demonstrated that low shear stress modulates VSMC proliferation and migration via the Caveolin-1-ERK1/2 pathway [62]. Increased blood flow stress in the vessel wall activates mechanical signaling pathways, triggering arterial remodeling to maintain stability, and impaired mechanical signaling and insufficient vascular remodeling contribute to the progressive expansion and eventual rupture of AAA [63]. At the same time, stimulation of endothelial cells on the vascular wall induces the release of molecules like nitric oxide (NO) and PGI2, which regulate VSMC contraction [64]. Although some studies have utilized computational fluid dynamics (CFD) and agent-based models (ABMs) to develop multiscale models that simulate vascular remodeling over time under different hemodynamically induced low-wall shear stress conditions, further investigation is needed [65]. In vivo, shear stress is dynamic, particularly in the arterial system, where pulsatile shear stress caused by the cardiac cycle exerts complex effects on VSMCs [66]. Most current studies rely on constant shear stress, highlighting the need for improved methods to simulate and analyze dynamic shear stress effects. Further elucidation of these intrinsic mechanisms could identify novel targets for preventing and treating vascular remodeling in AAA progression.

#### 3.2.3. Mitochondrial Energy Supply

Mitochondria, the primary organelles for energy metabolism, are closely linked to the contractile force of VSMCs [67]. Mitochondrial function plays a continuous and essential role in supporting actin–myosin interactions during VSMC contraction. In contractile VSMCs of the medial layer, mitochondria are moderately abundant, morphologically regular, and densely packed, typically positioned near the contractile filaments to form localized “energy zones”, where they are often anchored to the cytoskeleton to support stable structural organization and primarily generate ATP via oxidative phosphorylation (OXPHOS) to sustain contractile activity [68,69]. Under pathological conditions, mitochondrial fragmentation occurs, OXPHOS efficiency declines, ATP production is reduced, and contractile force and vascular compliance are subsequently impaired [69]. In contrast, migratory VSMCs located in the intimal layer exhibit increased mitochondrial abundance, more of a dynamic morphology, and functional specialization toward ROS production and calcium buffering, with single-cell transcriptomic data revealing the elevated expression of mitochondrial stress-related genes such as PGC1α and UCP2, indicating a functional divergence from medial VSMCs [70]. At the adventitia–media border, VSMC-like cells display a more scattered mitochondrial distribution and show a metabolic shift toward glycolysis and immune modulation [68].

A single-cell sequencing study revealed widespread mitochondrial dysfunction among aortic VSMCs, representing a prominent hallmark of aneurysmal disease [70]. Prohibitin 2 (PHB2), a key member of the prohibitin (PHB) family primarily localized in the mitochondrial inner membrane, is a multifunctional protein essential for maintaining the mitochondrial morphology and function, and it acts as a critical regulator of intracellular homeostasis and cell differentiation [71,72]. Recent studies suggest that targeting the PHB2-hnRNPA1-PKM2 axis to regulate VSMC energy metabolism could facilitate the treatment of cardiovascular diseases [71]. Jia and colleagues reported that VSMCs of PHB2^SMCKO^ mice exhibited a loss of their normal contractile phenotype, reduced contractile proteins, and increased ECM degradation compared to those of PHB2^Flox/Flox^ mice [73]. These findings indicate that VSMC function is dependent on energy supplied by mitochondria. In AAA, mitochondrial damage impairs energy production, notably reducing ATP synthesis and activating oxidative stress, all of which severely affect VSMCs, including their capacity to maintain the vascular structure, repair damage, and synthesize ECM [72,74].

## 4. Inflammation, Oxidative Stress, and Mitochondrial Dysfunction of the Aortic Wall and VSMCs

**Figure 3:**
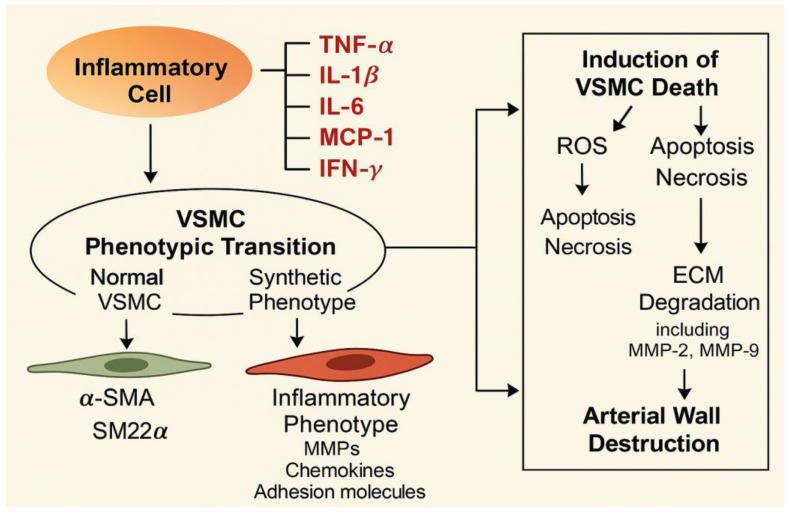
The mechanism by which inflammatory cells and their secreted cytokines (TNF-α, IL-1β, IL-6, MCP-1, and IFN-γ) affect VSMCs during the development of AAA. Inflammatory cells release cytokines that induce phenotypic transformation of normal VSMCs—from a contractile phenotype characterized by the expression of α-SMA and SM22α to a synthetic or even inflammatory phenotype. Inflammatory VSMCs produce MMPs, chemokines, and adhesion molecules, which further exacerbate local inflammation. At the same time, cytokines promote VSMC death (including apoptosis and necrosis induced by reactive oxygen species, ROS), and the contents released by dying cells contribute to ECM degradation, particularly through MMP-2 and MMP-9. This ultimately leads to structural destruction of the arterial wall and the formation and progression of AAA. Overall, the diagram reflects a pathogenic feedback loop between inflammation and VSMCs. Abbreviations: AAA: abdominal aortic aneurysm; VSMCs: vascular smooth muscle cells; MMPs: matrix metalloproteinases; ECM: extracellular matrix; ROS: reactive oxygen species.

Inflammation plays a crucial role in AAA progression, serving as both a driver and consequence of vascular injury. The inflammatory response involves multiple immune cell types, including macrophages, mast cells, neutrophils, dendritic cells, B cells, and T cells, all of which contribute to the AAA pathogenesis [75]. Endothelial cells and SMCs also participate in this process. Notably, VSMCs contribute to inflammation in several ways. VSMCs can produce pro-inflammatory cytokines to recruit immune cells to the vascular wall or be regulated by cytokines secreted by other cells [17]. Specifically, although VSMCs are not typically inflammatory cells, they can be activated under stress conditions such as aging, hypertension, and atherosclerosis, acquiring macrophage-like functions by secreting pro-inflammatory cytokines (IL-6, IL-1β, IL-10, MCP-1, TNF-α, and so on) [16]. These cytokines and chemokines recruit inflammatory cells to the aortic wall, exacerbating local inflammation and promoting AAA progression [17] (Figure 3). Studies using autologous cellular debris (CD) from late-stage AAA tissues to stimulate VSMCs revealed strong NF-κB activation, the activation and release of inflammasomes such as AIM2 and NLRP3, and the secretion of various pro-inflammatory factors, highlighting the crucial role of NF-κB in AAA-related inflammation in VSMCs [76]. Additionally, activation of signaling pathways such as mTOR, JAK/STAT, TGF-β/Smad, smooth muscle-enriched Nox4, and SMARCD1 promotes VSMC inflammation and aneurysm progression, whereas pathways including PPARγ, RhoGAP, and Nrf2-Keap1 attenuate the pro-inflammatory VSMC phenotype, thereby mitigating vascular inflammation and preventing AAA development [17,75,77,78]. On the other hand, inflammatory factors can indirectly affect VSMC function by mediating ECM degradation, which intensifies VSMC inflammation. For instance, OPN produced by leukocytes has been shown to regulate macrophage infiltration, thereby promoting vascular inflammation and elastin degradation, in part by activating NF-κB signaling, which subsequently stimulates MMPs to degrade matrix proteins [79]. Histopathological analysis of human aortic aneurysm tissues reveals extensive inflammatory cell infiltration, where these cells secrete pro-apoptotic factors that induce programmed cell death in VSMCs [80]. These findings illustrate the complex interactions between inflammation-related signaling pathways and inflammatory factors in regulating VSMCs’ behavior during AAA progression. Despite extensive research, current studies largely emphasize late-stage inflammation in AAA, with limited insight into the stage-specific evolution of VSMC inflammation. As the inflammatory response likely varies across disease stages, understanding these dynamics could inform the optimal timing for targeted therapies. Additionally, existing research primarily centers on the autonomous role of VSMCs in inflammation, with limited exploration of interactions between VSMCs and other cell types (e.g., macrophages, T cells, endothelial cells). The regulatory mechanisms of VSMC–immune cell interactions mediated by chemokines, cytokines, and exosomes remain insufficiently understood. These interactions may amplify inflammatory responses and accelerate AAA progression. A comprehensive understanding of these complex intercellular interactions could support the development of effective therapeutic strategies.

ROS and inflammation often activate each other, forming a positive feedback loop that plays a central role in VSMC dysfunction and AAA development. ROS originates from NADPH oxidases (particularly NOX1 and NOX4), the mitochondrial respiratory chain, and xanthine oxidase [81]. Inhibition of NADPH oxidase and iNOS attenuates ROS generation and consequently suppresses AAA progression, highlighting ROS inhibition in the treatment of AAA [82]. Low ROS levels may promote contractile function through signaling pathway activation, while excessive ROS lead to oxidative stress, mitochondrial dysfunction, and impaired VSMC contraction [83,84]. Meanwhile, NF-κB, a key regulator of inflammation, is widely activated in AAA [85,86]. Zhong et al. reported that elevated ROS levels in VSMCs from human and mouse AAA tissues lead to NF-κB pathway activation and production pro-inflammatory factors (IL-6, TNF-α) [86]. And extensive inflammatory infiltration mediated by T cells, macrophages, and B cells occurs in the aneurysmal wall, accompanied by alterations in the levels of ROS, IgM, IgG, CD38, GDF15, S100A4, and CD36 in the aneurysmal tissue of AAA patients [87]. This indicates that ROS can activate NF-κB, promoting the expression of pro-inflammatory cytokines such as IL-1β and TNF-α, which in turn further activate NOX enzymes and induce additional ROS production, forming a positive feedback amplification loop that leads to sustained VSMC dysfunction. Studies have also confirmed that the use of mitochondrial aldehyde dehydrogenase 2 (ALDH2) activators can reduce Ang II-induced ROS production, NF-κB activation, and apoptosis in human aortic smooth muscle cells (HASMCs), thereby alleviating aneurysm formation and limiting aortic dilation [88].

Mitochondrial damage and dysfunction are hypothesized to be actors in the altered production of ROS and oxidative stress [72]. The metabolic pathways and intrinsic mechanisms underlying VSMC mitochondrial dysfunction are complex and warrant further investigation. Glucose, lipid, and amino acid metabolism constitute the three primary metabolic pathways in VSMCs, playing a crucial role in vascular homeostasis regulation [89]. Studies indicate a strong correlation between energy loss and aortic diameter, with greater energy loss associated with more severe membrane degeneration, even in aortas of similar sizes [90]. Mitochondria are abundant in muscle cells and regulate most of the energy required for contraction [91]. Therefore, mitochondrial function is critical for metabolic homeostasis. Single-cell RNA sequencing revealed widespread mitochondrial dysfunction across various aortic cell types, representing a key pathological feature of aortic aneurysms [67]. Mitochondrial components and metabolic byproducts, as damage-associated molecular patterns (DAMPs), influence not only VSMC phenotypic switching but also play a role in amplifying inflammation [92]. Research has shown that DAMPs can be released into the cytoplasm or extracellular environment to promote inflammation, making mitochondrial damage in VSMCs a potential trigger for inflammation in AAA [92]. In VSMCs affected by AAA, mitochondrial dysfunction is prevalent, leading to disruption of the oxidative respiratory chain and impaired ATP production, which subsequently compromises the transmembrane H^+^ gradient and reduces mitochondrial membrane potential; this dysfunction also results in excessive ROS generation, causing mitochondrial DNA damage, mutations, and aberrant gene expression, thereby establishing a vicious cycle that exacerbates cellular impairment [93].

Energy metabolism in VSMCs is associated with metabolic reprogramming, with aerobic glycolysis and fatty acid β oxidation (FAO) being the main mitochondrial metabolic pathways for VSMCs [89,92]. Glucose transporter 1 (GLUT1), the main subtype in VSMCs, facilitates glucose uptake [94]. Studies on rat aortic smooth muscle cell lines (A7r5) and human vascular smooth muscle cell lines have shown that overexpression of GLUT1 results in a 44% increase in intracellular glucose concentration, enhancing the flow of glucose through glycolysis and the tricarboxylic acid (TCA) cycle, which in turn promotes VSMC proliferation [95,96,97]. Glycolysis also generates L-lactate, which serves as a substrate to boost mitochondrial reserve capacity, preventing an “ATP crisis” within cells and supporting VSMC proliferation [98]. Synthetic VSMCs exhibit increased glycolysis and decreased glucose oxidation [99,100]. In contrast, FAO produces more energy than glucose metabolism [101]. FAO involves the transport of long-chain fatty acids into the mitochondrial matrix via carnitine palmitoyltransferase 1 and 2 (CPT1 and CPT2), where they are oxidized to produce acetyl-CoA, NADH, and FADH2, generating energy [100]. It has been reported that octanoate enhances oxidative metabolism in the resting porcine carotid artery by increasing O_2_ consumption and TCA cycle anaplerosis (via glucose-dependent pyruvate carboxylation), compensating for inhibited aerobic glycolysis and maintaining high-energy phosphate levels, demonstrating fatty acids’ key role in vascular smooth muscle energy metabolism [102]. During phenotypic switching, synthetic VSMCs show reduced glucose oxidation and increased FAO, providing more energy to support rapid proliferation, migration, synthesis, and ECM secretion [100,103]. So, mitochondrial damage contributes to AAA development through various mechanisms, including inducing oxidative stress, promoting VSMC apoptosis and phenotypic switching, disrupting autophagy, causing energy metabolism imbalance, and exacerbating inflammation. Future therapies targeting mitochondrial metabolic pathways and small-molecule metabolites could offer promising approaches for preventing and treating AAA.

Emerging evidence highlights the critical role of metabolic dysregulation and metabolite-mediated inflammatory pathways in driving AAA progression and provides promising targets for diagnosis and therapy. Cui et al. employed untargeted metabolomics and mass spectrometry to identify and validate elevated plasma succinate levels in AAD patients compared to controls and individuals with other cardiovascular diseases [104]. Further validation in animal models demonstrated that macrophage-mediated inflammation upregulated this metabolite via the p38/CREB/OGDH axis [104]. Notably, p38 knockdown reduced succinate concentrations and alleviated AAD [105]. A recent study identified abnormal citrate accumulation in human and mouse aneurysmal tissues due to downregulation of ANK (a citrate membrane transporter) in VSMCs. ANK knockout in VSMCs induced cytosolic citrate accumulation and a citrate-related pro-inflammatory VSMC phenotype, finally leading to accelerated AAA formation [106]. Therefore, targeting ANK-mediated citrate transport in VSMCs may represent a novel diagnostic and therapeutic strategy for AAA. Additionally, beyond the correlation between metabolic changes in energy supply and AAA, Sun et al. reported that NR1D1, a metabolic nuclear receptor (NR), was upregulated in human and mouse VSMCs from AAA tissues, and NR1D1 knockout in mouse VSMCs suppressed AAA formation [107]. Subsequently, mechanistic studies revealed that NR1D1 deficiency restored the dysregulation and mitochondrial dysfunction of its direct transcriptional target ACO2 during early Ang II infusion prior to AAA formation, and that supplementation with α-ketoglutarate, a downstream ACO2 metabolite, prevented and treated AAA in mice in an NR1D1-dependent manner within VSMCs [107]. Moreover, as an emerging cytokine-related molecule, Gasdermin D (GSDMD) indirectly influences phenotypic switching. Gao et al., through single-cell transcriptome analysis, identified upregulation of gasdermin D (GSDMD), a pyroptosis effector, in aortic VSMCs during angiotensin II-induced AAA, and further demonstrated in animal models that GSDMD promotes ornithine decarboxylase 1 (ODC1) expression via ER stress–CHOP signaling, with elevated putrescine levels mitigating AAA development, suggesting that putrescine is a potential biomarker or therapeutic target [108]. These studies suggest the possibility of using metabolite-related products as biomarkers or potential targets.

## 5. Phenotypic Switching of VSMCs

**Figure 4:**
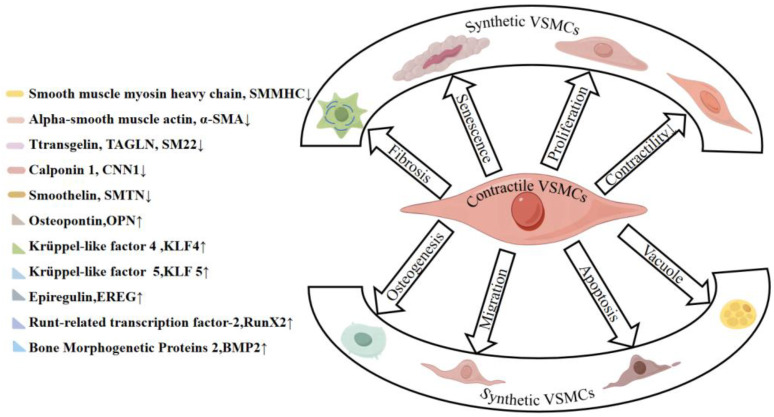
Illustration of VSMC contractile-synthetic switching during AAA progression. VSMCs exhibit high plasticity and can switch between two phenotypes to adapt to environmental changes. The differentiated VSMCs are in a “quiescent” state, expressing high levels of contractile proteins that enable stable smooth muscle contraction. Key markers of this phenotype include SMMHC, α-SMA, SM22, CNN1, and SMTN. The synthetic VSMCs, on the other hand, express low levels of contractile proteins but have high levels of molecules associated with proliferation, migration, fibrosis, and inflammation, such as OPN, EREG, KLF4, and BMP2. VSMCs may undergo transdifferentiation during disease progression or upon external stimulation, becoming more unstable phenotypic cells. Abbreviations: AAA: abdominal aortic aneurysm; VSMCs: vascular smooth muscle cells.

VSMCs show remarkable plasticity, enabling them to switch between distinct phenotypes to adapt to environmental changes. Differentiated VSMCs are typically in a “quiescent” state, expressing high levels of contractile proteins that facilitate stable smooth muscle contraction. Key proteins of this phenotype include α-SMA, SMMHC, SM22α, and CNN [15]. In contrast, synthetic VSMCs exhibit low levels of contractile proteins but express high levels of molecules related to proliferation, migration, fibrosis, and inflammation, such as OPN and epiregulin (EREG) (Figure 4). VSMCs can undergo transdifferentiation, acquiring a more unstable phenotype under external or internal stimuli in the development of AA and AD. This transdifferentiation impairs VSMC function, diminishing their capacity to synthesize the vascular matrix and consequently weakening the vascular wall [109].

VSMC phenotypic switching may lead to increased proliferation, migration, inflammation, or apoptosis, as discussed in other sections [110]. In addition, the phenotypic transformation of VSMCs is regulated by a variety of mechanisms, which we will review from the most studied cytokines, signaling pathways, and epigenetics, respectively.

### 5.1. Cytokines and Signaling Pathways

**Figure 5:**
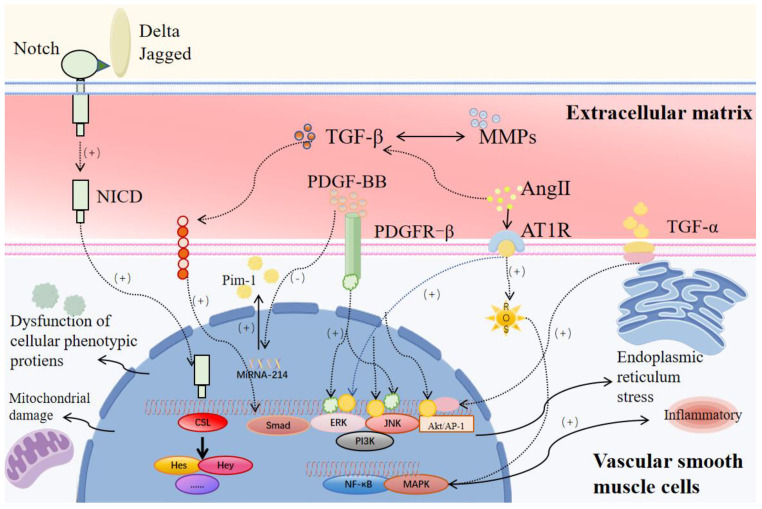
Molecular mechanisms of vascular smooth muscle cell phenotype switching during aneurysm formation. The phenotype switching of VSMCs is regulated by various cytokines and signaling pathways. Cytokines such as Ang II, PDGF-BB, TGF-β, and TGF-α can activate signaling pathways, including Notch, ERK/MAPK, PI3K/Akt, JAK/STAT, and TGF-β/Smad, which mediate intracellular ROS activation, inflammation, and endoplasmic reticulum stress, ultimately promoting phenotype switching. Abbreviations: VSMCs: vascular smooth muscle cells; TGF-β: transforming growth factor beta; ROS: reactive oxygen species; TGF-β: transforming growth factor beta; PDGF-BB: platelet-derived growth factor-BB; TNF-α: tumor necrosis factor alpha; ERK1/2: extracellular signal-regulated kinase 1/2; Ang II: angiotensin II; PI3K: phosphoinositide 3-kinase; Akt: protein kinase B; ERK: extracellular signal-regulated kinase; MAPK: mitogen-activated protein kinase; JAK: Janus kinase Janus; STAT: signal transducer and activator of transcription.

VSMC phenotypic switching in AAA is regulated by numerous signaling pathways (Figure 5). It is well established that signaling pathways in SMCs play a critical role in regulating various cellular functions, including contractility, proliferation, migration, phenotypic switching, apoptosis, and extracellular matrix remodeling, as supported by references cited in other sections of the manuscript. In terms of structural heterogeneity, SMCs in the aortic media primarily maintain a contractile phenotype, dependent on pathways such as TGF-β, MAPK/ERK, PI3K/Akt, and calcium signaling [111]. These pathways are essential for preserving vascular wall integrity, and their dysregulation can lead to SMC dedifferentiation, accelerated matrix degradation, and ultimately medial layer weakening, contributing to aneurysm formation [111]. In contrast, SMCs in the intima—most of which originate from medial SMCs that have migrated or proliferated in response to injury—tend to adopt a synthetic or pro-inflammatory phenotype. In this context, signaling pathways such as NF-κB, Notch, and Wnt/β-catenin become more active, driving inflammatory gene expression, extracellular matrix remodeling, and exacerbation of vascular pathology [112]. Since some of the cytokines and signal transduction pathways are covered elsewhere in the article, we will again provide an overview of a few core pathways:

#### 5.1.1. PDGF Pathway

PDGF is a common peptide regulatory factor that stimulates connective tissue proliferation and induces the division and proliferation of various cells, including VSMCs, fibroblasts, and glial cells [113]. PDGF-BB, a member of the PDGF family, functions as a potent mitogen, promoting VSMC proliferation and migration [114,115]. PDGF-BB is minimally expressed in normal vessels but is upregulated in cardiovascular diseases like atherosclerosis (AS) [116]. Han et al. demonstrated that PDGF-BB promotes VSMC phenotypic switching by binding to PDGFR-β and activating downstream ERK1/2 and JNK pathways, leading to a reduced expression of contractile markers such as α-SMA, CNN1, and SMMHC, increased secretion of molecules like OPN, and enhanced cell proliferation and migration, while small-molecule inhibitors targeting ERK1/2 effectively suppress this PDGF-BB-induced phenotypic transition [117]. PDGF-BB promotes VSMC phenotypic switching by downregulating MiRNA-214 and upregulating Pim-1, while MiRNA-214 inhibits this process by suppressing Pim-1 expression and EMT-mediated migration, with Pim-1 overexpression counteracting MiRNA-214’s effects via STAT3, AKT, and ERK signaling, contributing to coronary atherosclerosis progression [115]. Notably, a phenotype-based study on bone marrow mesenchymal stem cell-derived smooth muscle cells, selected for regenerating and repairing abdominal aortic aneurysms, revealed that the presence or absence of PDGF-BB in the growth medium influenced the development of a mature smooth muscle phenotype [118]. This phenomenon differs from PDGF-BB-induced phenotypic conversion. Most research on PDGF-BB and VSMC phenotypic switching focuses on cardiovascular diseases like AS or pulmonary arterial hypertension (PAH), leaving its role and specific molecular mechanisms in AAA requiring further exploration [119].

#### 5.1.2. TGF-β/Smad Pathway

Inside the cell, TGF-β initially associates with latency-associated peptide (LAP) and covalently binds to latent transforming growth factor-beta binding proteins (LTBPs), forming an inactive complex that is secreted into the extracellular matrix, and proteolytic degradation of LTBP triggers LAP dissociation from TGF-β, enabling free TGF-β to bind its receptors and exert physiological effects [120]. In the human body, TGF-β primarily exists in three isoforms with distinct tissue-specific expression patterns: TGF-β1 is present in epithelial cells, smooth muscle cells, hematopoietic cells, and fibroblasts; TGF-β2 in epithelial cells and neurons; and TGF-β3 mainly in mesenchymal cells [121]. TGF-β1 mainly binds to type I (TGFBR1) and type II (TGFBR2) receptors, activating the canonical Smad-dependent pathway to regulate the contractile phenotype of VSMCs [122,123,124,125]. Additionally, TGF-β1 activates non-Smad pathways, such as RhoA-GTP, which in turn activates ROCK, leading to myosin phosphorylation, increased contractility, and vascular tone maintenance [126]. TGF-β1 has also been reported to regulate VSMC mechanical contractility by modulating actin cytoskeleton reorganization via ERK1/2 [127]. Under physiological conditions, the TGF-β pathway promotes SMC contraction by upregulating contractile proteins, including SMMHC, α-SMA, transgelin (TAGLN, SM22), calponin 1 (CNN1), and smoothelin (SMTN) [123,126,128,129,130]. But it can also induce vascular stiffness and dysfunction in pathological states [15,131]. Excessive activation or abnormal signaling of TGF-β1 prompts VSMCs to transition to a synthetic phenotype, reducing their contractile ability and promoting ECM overproduction via classical and non-classical TGF-β pathways, including TGF-β/smad, MAPK (ERK1/2, p38 MAPK, JNK), NF-κB, and PI3K/Akt, which collectively contribute to vascular stiffness, dysfunction, decreased compliance, and the development of cardiovascular diseases such as hypertension, atherosclerosis, and aneurysms [50,132,133,134,135,136]. Tingting et al. demonstrated that TGF-β neutralization exacerbates angiotensin II-induced TAA and AAA [137]. Consequently, disruption of TGF-β receptors in VSMCs impairs contractile function and promotes aneurysm formation. Furthermore, Da et al. demonstrated that AGGF1 enhances the interaction between its receptor, integrin α7, and LAP-TGF-β1, preventing LAP-TGF-β1 cleavage into its mature form, and this interaction suppresses Smad2/3 and ERK1/2 phosphorylation in VSMCs, mitigating thoracic aortic aneurysm progression [128]. Similarly, Shi and Yaning et al. found that inhibition of the TGF-β1/ERK1/2/CTGF signaling pathway alleviated atherosclerosis and restenosis by preventing phenotypic transition, proliferation, and migration of VSMCs and maintaining vascular tone [138]. Thus, TGF-β1 activation is closely associated with the stability of the VSMC contractile phenotype. Investigating the distinct roles of classical and non-classical TGF-β pathways in aneurysm formation may reveal novel therapeutic targets for AAA.

#### 5.1.3. Notch Signaling Pathway

The Notch signaling pathway is a highly conserved cell-to-cell communication system involving Notch receptors (Notch1, Notch3) and ligands (Jagged1, Dll4). Upon activation, the Notch receptor releases the Notch intracellular domain (NICD) into the nucleus, where it regulates gene expression and influences cell proliferation, differentiation, and survival [139]. In VSMCs, Notch2 and Notch3 receptors, along with the ligand Jagged1, are predominant [139,140]. The Notch signaling pathway has been found to be activated in human and mouse abdominal aortic aneurysm tissues [141]. Concomitant pharmacological inhibition of the Notch signaling pathway inhibits the progression of abdominal aortic aneurysms [141,142,143]. Notch signaling coordinately regulates three key cell types in AAA pathogenesis: endothelial cells, immune cells, and VSMCs. Specifically, Notch signaling in endothelial cells regulates vascular homeostasis and barrier integrity [67,75,144,145]; in immune cells, it modulates inflammatory responses and macrophage polarization [67,75]; and in VSMCs, it influences phenotypic switching, proliferation, and apoptosis [67]. Dysregulation of Notch signaling in these cell types has been implicated in extracellular matrix degradation, inflammation, and aortic wall weakening—key pathological features of AAA. And Notch activation contributes to vascular remodeling by promoting VSMC proliferation and migration [146]. In terms of mechanisms, firstly, Pan et al. demonstrated that the CBF1 binding site in the renin gene promoter responds to Notch1 ICD-induced transcriptional activation, suggesting that the Notch signaling pathway serves as an upstream regulator of VSMC contraction, vascular tension, and phenotype [147]. Moreover, Tang and his colleagues demonstrated that inhibition of Notch-induced smooth muscle α-actin expression by interfering with the interaction of the Notch intracellular domain with the binding site [148]. Secondly, Notch signaling directly regulates renal vascular tone, as evidenced by Boulos et al., who found that in Notch3-deficient mice, renal vascular resistance failed to increase in response to vasoconstrictors as expected, highlighting Notch’s role in tone regulation [149]. By regulating vascular tone, the Notch signaling pathway alters the VSMC phenotype under pathological conditions [149]. The evidence demonstrates that the Notch signaling pathway exhibits a dual role in VSMC phenotypic switching, promoting or inhibiting the switch based on the pathological conditions.

#### 5.1.4. Ang II/AT1R Signaling Pathway

Angiotensinogen, primarily produced by the liver, is cleaved by renin in the glomeruli to generate Ang I. Ang I then binds to angiotensin-converting enzyme (ACE), which is secreted by vascular endothelial cells, and is subsequently converted into Ang II in the bloodstream—this constitutes the classical Ang II production pathway [150,151]. The alternative pathway, independent of renin, involves enzymes such as angiotensinase and cathepsin, which can directly convert angiotensinogen to Ang I or Ang II [152,153]. Renal ischemia and activation of the renin–angiotensin system (RAS) are the primary triggers for renin activation [153]. ACE is a zinc metalloproteinase primarily expressed in pulmonary vascular endothelial cells but also present in tissues such as the uterus, placenta, heart, blood vessels, kidneys, brain, and adrenal cortex. ACE catalyzes the conversion of Ang I to Ang II, a potent vasoconstrictor, while also degrading bradykinin, a vasodilator, thereby regulating blood pressure and cardiovascular homeostasis [154]. As the primary effector molecule in the RAS pathway, Ang II functions by activating two G protein-coupled receptors: AT1R and AT2R [155,156,157]. AT1R is widely expressed in various tissues and organs, including the heart, blood vessels, kidneys, and brain. When Ang II binds to AT1R, it induces vasoconstriction, inflammation, and elevated blood pressure [158]. Thus, AT1R is regarded as a pro-inflammatory and pro-hypertensive receptor. In contrast, AT2R is primarily expressed in fetal tissues but is also present in certain adult tissues, including the brain, blood vessels, and heart. Upon activation, AT2R promotes vasodilation, exerts anti-inflammatory effects, and lowers circulatory pressure [158]. Thus, AT2R is recognized as an anti-inflammatory and antihypertensive receptor.

In 1999, Daugherty and Cassis demonstrated that Ang II promotes atherosclerosis in low-density lipoprotein receptor (LDLR) knockout mice. Later, they further discovered that Ang II also facilitates both atherosclerosis and AAA formation in apolipoprotein E (ApoE) knockout mice [159]. This discovery provided a foundation for subsequent research on the mechanisms underlying AAA development and the establishment of Ang II-induced AAA animal models. Subsequently, the role of the AngII/AT1R signaling pathway in AAA-VSMCs has been gradually clarified, and the current research has found that the binding of Ang II to AT1R can active ERK/MAPK, PI3K/Akt, and JAK/STAT pathways, shifting VSMCs from a contractile to a synthetic phenotype [160,161,162]. Additionally, Ang II/AT1R activates NADPH oxidase, resulting in ROS generation, which critically mediates VSMC phenotypic switching via oxidative stress [162]. This pathway destabilizes the ECM through TGF-β and MMP regulation, increasing VSMCs’ susceptibility to phenotypic switching [162,163].

Angiotensin II receptor blockers (ARBs), traditional drugs that target the Ang II/AT1R signaling pathway, are controversial for their efficacy in relieving AAA. However, studies have shown that ARBs may slow the progression of AAA by inhibiting the binding of Ang II to its type I receptor (AT1R). For example, one study found that ARBs were able to limit the growth of AAA [164]. Hackam et al. demonstrated that patients using ACE inhibitors had a lower risk of AAA rupture [165]. At the same time, a randomized placebo-controlled trial found that ACE inhibitors moderately slowed the growth of small AAA compared to a placebo but did not significantly lower blood pressure [166]. A national cohort study in Denmark indicated that treatment with ACE inhibitors or ARBs was moderately associated with reduced mortality in AAA patients but showed no benefit in surgical outcomes [167]. However, a prospective cohort study across 93 UK hospitals reported faster aneurysm growth in patients using ACE inhibitors. This finding contradicts data from a large Canadian database, which suggested a lower risk of aneurysm rupture in ACE inhibitor users [168]. Some evidence suggests no association between ACE inhibitor or ARB use and AAA growth. However, ACE inhibitors are linked to a lower risk of AAA rupture and related events [169]. Given these conflicting findings, several large randomized controlled trials are currently underway. Additionally, studies in AAA mouse models support the potential benefits of this strategy [170,171,172,173,174].

#### 5.1.5. Inflammation-Related Cytokines

Inflammation-related factors are closely related to the phenotypic switching of VSMCs, and some of them have been described in the chapter on inflammation. TNF-α, along with numerous inflammatory cytokines, is significantly upregulated following vascular endothelial cell damage and can induce VSMC proliferation, migration, and phenotypic switching. Tanaka H and colleagues identified a highly proliferative VSMC subgroup producing elevated TNF-α levels in a rabbit abdominal aortic balloon injury model [175]. In vitro studies by Chou and colleagues confirmed that TNF-α enhances VSMC migration, while inhibition of the Akt/AP-1 signaling pathway blocks both TNF-α-induced phenotypic switching and cell migration [176]. Recent clinical research confirmed elevated TNF-α levels in intracranial aneurysm (IA) patients. Silencing TNF-α prevented VSMC apoptosis and phenotypic switching, improving IA progression [177]. These findings further confirm the relationship between TNF-α and VSMC phenotypic changes. In animal studies, Wen and colleagues found that injecting umbilical cord mesenchymal stem cells (UC-MSCs) into AAA model mice suppressed TNF-α expression and restored the contractile phenotype of VSMCs [78]. These studies indicate that within the disease microenvironment, increased TNF-α secretion drives VSMC phenotypic switching. Targeting TNF-α secretion and expression may offer a promising strategy to slow cardiovascular disease progression, as other inflammatory mediators such as IL-1β, IL-6, IL-8, and MCP-1 not only drives VSMC phenotypic transformation but also correlates strongly with AAA progression, highlighting the need for deeper insight into their mechanisms to identify novel therapeutic targets [118].

### 5.2. Epigenetic Modifications

Epigenetic modifications—including DNA methylation, histone modifications, non-coding RNA regulation, and chromatin remodeling—are emerging as key focuses in advancing VSMC phenotypic switching research.

#### 5.2.1. DNA Methylation

DNA methylation, a classical epigenetic modification, typically occurs at CpG islands in gene promoter regions, leading to gene expression inhibition. It has been demonstrated that 5-aza-2’-deoxycytidine (a DNA methylation inhibitor) or DNA methyltransferase 1 knockout reduced 5-methylcytosine levels in VSMCs, preventing excessive dedifferentiation, proliferation, and migration [136,178]. Atherosclerosis is a risk factor for AAA, potentially linked to AAA through complex inflammatory mechanisms and pathological alterations in the vascular wall [179]. As the pathological hallmark of atherosclerosis, endothelial dysfunction contributes to increased vascular permeability while demonstrating significant associations with dysregulated DNA methylation [180]. Subsequently, a number of clinical and basic studies have found that changes in the DNA methylation level of homocysteine (Hcy) in serum can promote the expansion rate or growth rate of AAA [181,182,183,184]. In addition, Toghill BJ et al. demonstrated gene-specific alterations in DNA methylation within VSMCs derived from AAA patients, identifying significant associations between CpG methylation in the SMYD2 promoter and reduced SMYD2 expression, thereby indicating the presence of aberrant DNA methylation in AAA-related VSMCs [185]. DNA methylation critically influences VSMC protein synthesis and phenotype maintenance by regulating the methylation status of key gene promoters. In healthy medial VSMCs, promoters of contractile markers such as α-SMA, SM-MHC, and calponin exhibit low methylation, supporting their high expression and preserving the contractile phenotype [136,186]. In contrast, under pathological conditions such as AAA, increased activity of DNA methyltransferases (e.g., DNMT3B) leads to hypermethylation of these contractile gene promoters, resulting in transcriptional repression and promoting the phenotypic switch of VSMCs toward a synthetic or inflammatory state [186]. Concurrently, pro-inflammatory genes such as OPN often undergo promoter hypomethylation, resulting in their upregulation and contributing to extracellular matrix degradation and vascular wall remodeling [187,188].

#### 5.2.2. Histone Modifications

Histone modifications influence chromatin openness or condensation through acetylation, methylation, phosphorylation, and other alterations on specific histone tail residues, thereby regulating gene transcription [189,190]. Histone acetylation refers to the enzymatic process by which histone acetyltransferases (HATs) catalyze the addition of acetyl groups to histone proteins, specifically targeting lysine residues on the histone tails [189]. Previous studies have shown that histone acetylation and the expression of lysine K histone acetyltransferases (KATs) are elevated in human AAA tissues [191]. However, in the same year, it was proposed that reversible enzymes involved in the regulation of histone acetylation post-translational modifications (PTMs) were significantly upregulated in AAA [191]. Specifically, histone deacetylases (HDACs) and HATs corresponding to KATs were implicated. In mouse models, HDAC inhibitors were observed to limit aneurysm progression [192]. It has been demonstrated that HDAC inhibitors can suppress the expression of matrix metalloproteinases MMP2 and MMP9 in VSMCs derived from AAA mouse models, thereby reducing the incidence of AAA [193]. These findings indicate that HDACs may contribute to ECM degradation, elevated inflammatory mediator levels, and VSMC apoptosis [192]. In addition, there are novel findings regarding histone methylation. SUMO-specific protease 1 (SENP1), a cysteine protease, regulates VSMC phenotypic switching by modifying the serum response factor (SRF) via small ubiquitin-related modifiers (SUMOs) and facilitating the SRF-ELK1 complex formation [194]. The ELK2 inhibitor AZD6244 effectively blocked this process, reducing phenotypic transformation and alleviating cardiovascular diseases [194]. Furthermore, the mechanism of histone modifications in the cardiovascular field, including AAA, is still being studied [195]. It is believed that the research on these histone modifications will play a more enlightening role in the study of the mechanism and treatment of AAA disease progression.

#### 5.2.3. Chromatin Remodeling Complexes

Additionally, chromatin remodeling complexes alter DNA–histone interactions, affecting gene availability. The SWI/SNF complex modulates chromatin to regulate VSMC switching [196]. Furthermore, BAF60a, a component of the SWI/SNF complex, was upregulated in human and experimental mouse abdominal aortic aneurysm lesions, and genetic ablation of BAF60a in VSMCs was shown to attenuate AAA formation by inhibiting inflammation and extracellular matrix degradation in AAA mouse models induced by both Ang II infusion and elastase perfusion [197]. Further study confirmed the crucial role of BAF60c in maintaining VSMC homeostasis, underscoring its therapeutic potential for AAA prevention and treatment [198] and indicating the essential role of chromatin remodeling in preserving VSMC homeostasis and preventing AAA.

#### 5.2.4. Non-Coding RNA in Abdominal Aortic Aneurysms

MicroRNAs (MiRNAs) are highly conserved, 17–23-nucleotide-long, non-coding, single-stranded RNA molecules in eukaryotes that inhibit signal transduction by degrading mRNA. Various MiRNAs have been demonstrated to regulate VSMC function, including MiRNA-21, MiRNA-29b, MiRNA-24, MiRNA-103a, MiRNA-143/145, MiRNA-205, MiRNA-712, MiRNA-15, MiRNA-181b, MiRNA-221/222, and MiRNA-126/146a (Table 1).

Yang et al. observed a significant increase in MiRNA-26a expression in VSMCs cultured under PDGF-BB stimulation and in arteries with neointimal lesions, and they demonstrated that MiRNA-26a regulates VSMC phenotypic switching by target gene Smad1 [212]. Additionally, endothelial-derived MiRNA-92a influences the VSMC phenotype, promoting atherosclerosis or preventing in-stent restenosis [213,214]. MiRNA-23b expression decreases in Ang II-treated ApoE^−/−^ mice and human AAA-VSMC tissue, accelerating the suppression of contractile markers [201]. Recent studies have identified additional non-coding RNAs, including circMAP3K5, circDcbld1, and circLrp6, as key regulators of VSMC phenotypic transformation [215,216,217]. Overall, non-coding RNA contributes to AAA formation by partially inhibiting or promoting VSMC phenotypic switching.

## 6. VSMC Degradation

Research confirms that apoptosis of abdominal aortic VSMCs reduces cell numbers, leading to structural arterial changes, impaired function, and aortic expansion, which critically contributes to AAA progression [218]. Lopez et al. observed the medial VSMC morphology in AAA using light microscopy, identifying membrane shrinkage, chromatin condensation, and apoptotic body formation [219]. At the same time, VSMC aging and depletion are notable pathological features in advanced stages of aneurysms [17]. Multiple mechanisms regulate VSMC apoptosis and contribute to AAA progression, presenting potential therapeutic targets. Recent research indicates that the anti-apoptotic protein cartilage oligomeric matrix protein (COMP) derived from thoracic periaortic adipose tissue (T-PVAT) exerts anti-apoptotic effects on VSMCs and mitigates AAA formation [220]. Wang et al. demonstrated that glutamine prevents AAA in mice by inhibiting VSMC apoptosis, suppressing M1 macrophage activation, reducing oxidative stress, and preventing extracellular matrix degradation [221]. Another study found that Ang II stimulated increased levels of MMP-2 and MMP-9 in VSMCs, decreased elastin expression, and promoted apoptosis, autophagy occurrence, and secretion of inflammatory factors, while resveratrol (RES) pretreatment improved this effect [222]. Wen et al. demonstrated that ATF3, a key transcriptional regulator in cardiovascular disease, suppresses advanced mitochondria-dependent apoptosis by upregulating its direct target BCL2, highlighting ATF3 as a potential therapeutic and prognostic marker in AAA [223]. Evidence has shown that XIST, a key regulator of mammalian X chromosome inactivation, induces arterial smooth muscle cell apoptosis via the miR-29b-3p/Eln axis, thereby accelerating thoracic aortic aneurysm progression [224]. Zhang et al. further confirmed that XIST inhibition attenuates AAA in mice by suppressing VSMC apoptosis through modulation of the miR-762/MAP2K4 axis [225]. Their findings reveal a novel ceRNA circuit that regulates key factors in AAA pathogenesis. Analysis of VSMCs from AAA patients has revealed significant upregulation of apoptosis and aging-related proteins such as p21 and p16. Interestingly, nicotinamide phosphoribosyltransferase was found to reverse the aging phenotype of VSMCs, thereby inhibiting AAA progression [226]. Lu et al. reported that the expression of the autophagy regulator transcription factor EB (TFEB) was significantly downregulated in aneurysmal samples from both humans and mice. Treatment with 2-hydroxypropyl-β-cyclodextrin (2HPβCD) increased B-cell lymphoma-2 (BCL-2) expression in a TFEB-dependent manner, inhibiting VSMC apoptosis induced by β-aminopropionitrile and Ang II, thereby mitigating AAA formation and progression [227].

In addition to apoptosis, other types of cell death are also involved, such as PANoptosis, autophagy, ferroptosis, and cuproptosis. PANoptosis is identified as a novel inflammatory cell death pathway integrating features of pyroptosis, apoptosis, and necroptosis, regulated by the PANoptosome complex. Li et al. [228] reported the upregulation of ZBP1 and AIM2, key PANoptosis markers, in AAA tissues and Ang II-treated VSMCs. Combined stimulation with TNF-α and IL-1β intensified VSMC death, while their neutralization reduced inflammation and aneurysm formation. Separately, Malireddi et al. [229] demonstrated that inflammatory mediators such as IL-1β, TNF-β, IFN-γ, and TNF-α, as well as aging MCP-1, IL-6, and MMP2 released by aging VSMCs, contribute to PANoptosis-driven VSMC degradation in AAA. Recent studies have shown that cryptotanshinone (CTS), a well-known herbal compound, prevents abdominal aortic aneurysm formation by targeting smooth muscle cells through the Keap1-Nrf2-GSDMD-pyroptosis axis via its potent anti-inflammatory properties. Similarly, other studies have identified novel targets for AAA mitigation through anti-inflammatory pathways and inhibition of aortic smooth muscle cell pyroptosis [108,230,231,232,233,234]. Additionally, ferroptosis represents a novel target for alleviating AAA [235]. VSMC ferroptosis is confirmed in both human and mouse AAA tissues [236]. A major risk factor of AAA, cigarette smoking has been proven to induce ferroptosis in VSMCs [237]. Inhibition of VSMC ferroptosis by Ferrostatin-1 prevented AAA formation in mice [238,239,240]. A sialic acid-containing type of glycosphingolipid named GM3 was shown to suppress lipid peroxidation and reduce iron deposition, thereby inhibiting VSMC ferroptosis and reducing AAA incidence [235]. These studies demonstrate the participation of VSMC ferroptosis in AAA and highlight the potential application of ferroptosis inhibitors in treating AAA. In addition, cuproptosis is a newly discovered form of regulated cell death triggered by excessive intracellular copper, distinct from apoptosis, necrosis, ferroptosis, and other known cell death pathways [104]. Multi-omics and machine learning studies reveal cuproptosis-related genes (e.g., PIM1, DLD) as key players in AAA pathogenesis [241,242,243], implicating copper-induced cell death in VSMC dysfunction and extracellular matrix remodeling. However, the exact role of cuproptosis in AAA, especially in VSMCs, warrants further experimental validation to establish causal mechanisms and translational relevance.

## 7. Degradation of ECM by VSMCs

VSMCs are involved in the regulation of aortic wall elasticity and the production of the ECM [14]. Normally, VSMCs, together with ECM proteins, form functional units that are essential for maintaining structural and functional integrity [16]. During AAA development, VSMCs produce various MMPs, leading to ECM degradation in the aortic wall. ECM degradation disrupts VSMC proliferation, adhesion, and migration, leading to phenotypic changes and apoptosis. This initiates a vicious cycle of VSMC loss and further ECM degradation, ultimately promoting AAA formation [244]. ECM degradation is mediated by various proteases, including the MMP family and metalloproteinase (ADAM) family [14,183].

MMPs are zinc-dependent endopeptidases that degrade key ECM components, including collagen (types I, III, IV), elastin, laminin, and proteoglycans. Notably, MMP-1, MMP-8, and MMP-13 function as collagenases, breaking down types I and III of collagen, thereby compromising the structural integrity of the aortic wall [245,246,247,248]. Gelatinases, including MMP-2 and MMP-9, exacerbate damage to the vascular basement membrane and ECM by degrading type IV collagen and gelatin [249,250,251,252]. Additionally, enzymes such as MMP-12 primarily degrade elastin, weakening the arterial walls and promoting aneurysm formation [253]. MMPs not only coordinate ECM remodeling but also regulate VSMC proliferation, migration, and apoptosis, as well as the recruitment and behavior of inflammatory cells. For instance, an in vitro study by Ramella et al. found that suppression of MMP-9 in endothelial cells reduced stromal protease levels in VSMCs and inhibited TNF-α-mediated NF-κB activation [248,251,254]. This conclusion is confirmed by other studies [167,170]. Previous studies in humans and rodents have shown a significant increase in MMP synthesis in AAA-VSMCs compared to the control group, particularly MMP-2 and MMP-9 [247,250,251,252,255]. Additionally, MMPs such as MMP-1, MMP-3, MMP-9, MMP-10, MMP-12, and MMP-13 are also expressed in AAA and TAA tissues [245,246,256,257,258,259]. MMPs are regulated by tissue inhibitors of metalloproteinases (TIMPs). Studies have found that TIMP expression is reduced in AAA and TAA tissues [188,260,261,262,263]. This indicates that the secretion and activation of MMPs in AAA and TAA tissues are regulated by TIMP.

In addition, a significant positive correlation between ADAM and VSMCs was observed in human abdominal aortic aneurysm tissue [183]. ADAM and ADAMTS belong to the adamalysin subfamily of the metallopeptidase family [264]. Most ADAMs are membrane-bound enzymes that regulate proteolytic enzymes and intercellular adhesion via their disintegrin domains [265]. ADAMTS is a soluble zinc protease with a molecular structure similar to ADAM, but it contains a different number of thrombospondin (TSP) motifs. To date, 37 ADAM species have been identified in rats, 34 in mice, and 21 in humans, with 19 members classified by their known substrates, 13 of which are proteolytically active [266,267]. In rodent studies, Liang et al. found elevated expression of ADAM10, ADAM17, MMP-2, and MMP-9 in a rat model of TAA established by calcium chloride [268]. ADAM15 plays a key role in abdominal aortic aneurysms by regulating VSMC function. ADAM15 deficiency exacerbates Ang II-induced aortic remodeling, leading to abdominal aortic aneurysm [269]. Similarly, ADAMTS5 catalytic domain deletion has been shown to promote TAA expansion [270]. Another study found that ADAM10 alleviates AAA by inhibiting the HMGB1/RAGE/NF-κB signaling pathway and MMP activity [271]. However, it is controversial that recent studies have confirmed that inhibition of ADAM10 expression will improve AAA formation and incidence [272,273]. In animal models of systemic ADAMTS1 knockout and ADAMTS1 haploinsufficiency, the former reduces the rupture rate of the thoracic aorta and dissection, whereas the latter is associated with a higher incidence of AA or fatal aortic dissection [274,275]. These studies suggest that each ADAM or ADAMTS may have distinct or even opposing effects on aneurysm severity, and that complete inhibition of these proteases may not be an appropriate treatment. Targeting specific ADAM or ADAMTS in distinct cell populations or at strategic time points during disease progression may offer more effective therapeutic options.

## 8. Conclusions and Future Perspective

This review summarizes the current research progress on VSMCs in the context of AAA, which may aid in preventing the condition and developing effective treatment strategies. However, several unresolved issues remain. Firstly, the origin of the VSMCs involved in the disease process is often uncertain, making it crucial to identify the source of pathological VSMCs. Secondly, current research typically treats VSMCs as a homogeneous group, overlooking their heterogeneity at different stages or in various pathological regions of AAA. While some studies suggest that VSMCs begin to exhibit abnormalities in the early stages of AAA, definitive evidence is lacking. VSMCs may display different phenotypes and functions at different stages of AAA, an important issue that future research must address. Thirdly, in cardiovascular research, metabolic indicators such as energy metabolism, lipid metabolism, and enzymes involved in aerobic glycolysis have been highlighted. However, few studies have explored the regulation of VSMCs by metabolic pathways (e.g., mitochondrial function, glucose metabolism) or the influence of nutritional status (e.g., cholesterol levels). Metabolic dysregulation in AAA may significantly impact VSMC function and inflammatory responses. A lack of understanding in these areas could mean missed opportunities for critical intervention. Finally, while AAA predominantly affects elderly men, gender differences in the disease are an important research aspect that should not be overlooked. Sex hormones may play a significant role in VSMC phenotypic switching and inflammatory responses, yet this area remains relatively underexplored. Some studies have found that female mice have lower rates of abdominal aortic aneurysm rupture and reduced smooth muscle cell degradation compared to male mice [276]. Androgen receptors have been shown to promote AAA development in male mice through IL-1 and TGF-β1 [276]. In vitro studies also indicate that phosphorylated Akt levels are higher in male rat aortic smooth muscle cells than in females, a factor that is crucial for AAA development [277]. Therefore, examining gender differences in VSMC dysfunction in AAA is essential. Ignoring these differences may lead to biased treatment strategies that do not offer the most effective interventions for both sexes.

In conclusion, significant challenges remain in elucidating the relationship between VSMCs and AAA. Multi-level mechanistic research, particularly at the molecular level, will provide valuable insights and guidance for future drug therapies.

## Figures and Tables

**Table 1:** Effect of microRNA on VSMCs in aortic aneurysm disease and its molecular mechanism.

RNA	Animal Model/Sample/Cell	Modeling Method	Downstream Molecule/ Signaling Pathway	Promotes (+)/Inhibits (−) VSMC Phenotypic Switching	VSMC Differentiation Markers	VSMC Dedifferentiation Markers	Other Cytokine/Gene	Disease	Ref.
MicroRNA-134-5p	Mice	High-fat Diet + Chronic AngII Infusion	STAT 5 B/ITGB 1 Pathway	Inhibition (−)	α-SMA SM22α CNN1 SMMHC	MMP2 MMP9 MMP12	ADAMTS1 ADAMTS4 ADAMTS7 Col2A1 VEGFA SMAD6 MKNK1	Thoracic aortic dissection	[199]
MicroRNA-342-5p	MOVAS cells	MOVAS cells were transfected with miR-342-5p mimics	Akt Pathway	Activation (+)	α-SMA	Vimentin	——	——	[200]
MicroRNA-23b	Mice	Chronic infusion of AngII on the backgroun of APOE knockout mice.	FoxO4 Pathway	Inhibition (−)	α-SMA SM22α CNN1	——	——	Abdominal aortic aneurysm	[201]
MicroRNA-126-5p	Mice	Chronic infusion of AngII on the background of APOE knockout mice.	VEPH1 Pathway	Activation (+)	α-SMA SMMHC	PCNA Vimentin	MMP2 MMP9	Abdominal aortic aneurysm	[78]
MicroRNA-29b	——	——	ATG14 Pathway	Inhibition (−)	α-SMA SM22α SM-MHC	——	MMP-2 MMP-3 TNF-α Beclin-1 ATG5 ATG14 p62 Beclin-1 ATG5 ATG14	Intracranial aneurysm	[202]
MicroRNA-128-3p	Mice	ApoE^−/−^ animals of either gender with a hypercholesterolemic diet for 16 weeks	KLF4 Pathway	Inhibition (−)	α-SMA CNN1 SMMHC SM22α	——	——	Atherosclerosis and carotid stenosis	[203]
MicroRNA-564	Mice	AngII infects cells	SKI/NRGN Pathway	Inhibition (−)	α-SMA MHC	——	——	Abdominal aortic dissection	[204]
MicroRNA-199a-5p-	AAA-VSMCs from patients		SIRT1/ROS Pathway	Activation (+)	α-SMA		P53 p21	Abdominal aortic aneurysm	[205]
MicroRNA-126	Mice	Carotid artery ligation	Argonaute2 Pathway	Inhibition (−)	α-SMA	——	——	Atherosclerosis	[206]
MicroRNA-24	Murine	Porcine-pancreatic-elastase (PPE) and AngII infusion	MAPK/NF-κB Pathway	Inhibition (−)	——	——	IL-6 IL-8 IL-1β TLR4	Abdominal aortic aneurysm	[207]
MicroRNA-221/222	Human samples	——	——	——	——	——	——	Acute coronary syndrome	[208]
MicroRNA-146a	Rat VSMCs	Artificially synthesized miR-146a mimics was transfected into cultured primary rat VSMCs in vitro	p53 Pathway	Activation (+)	——	CCK-8 Cyclin D1	Caspase-3 PTEN	——	[209]
MicroRNA-155-5p	HASMC	H_2_O_2_ or NaAsO_2_ suppressed viability and induced apoptosis of VSMCs	FOS/ZIC 3 Pathway	Activation (+)	——	Cyclin A Cyclin B Cyclin D	Caspase-3 Bcl-2	Abdominal aortic aneurysm	[210]
MicroRNA-195-5p	Rat VSMCs	Osteogenic induction of VSMCs by β-glycerophosphate (β-GP)	Wnt/β-catenin Pathway	Activation (+)	——	Runt Runx2 BMP2 ALP OCN Smad7	IL-6 TNF-α	——	[211]

## Data Availability

Not applicable.

## Abbreviations

AAA	Abdominal Aortic Aneurysm;
VSMCs	Vascular Smooth Muscle Cells;
TAA	Thoracic Aortic Aneurysm;
MMPs	Matrix Metalloproteinases;
ECM	Extracellular Matrix;
SMCs	Smooth Muscle Cells;
TGF-β	Transforming Growth Factor Beta;
ROS	Reactive Oxygen Species;
α-SMA	Alpha-Smooth Muscle Actin;
TAGLN	Transgelin;
SM22	Smooth Muscle Protein 22;
CNN1	Calponin 1;
SMTN	Smoothelin;
ECIS	Endothelial Cell-Isolation System;
LRP1	Low-Density Lipoprotein Receptor-Related Protein 1;
NO	Nitric Oxide;
PGI2	Prostacyclin;
cGMP	Cyclic Guanosine Monophosphate;
PHB	Prohibitin;
OPN	Osteopontin;
EREG	Epiregulin;
AD	Aortic Dissection;
PDGF-BB	Platelet-Derived Growth Factor-BB;
TNF-α	Tumor Necrosis Factor Alpha;
AS	Atherosclerosis;
SMMHC	Smooth Muscle Myosin Heavy Chain;
EMT	Epithelial–Mesenchymal Transition;
ERK1/2	Extracellular Signal-Regulated Kinase 1/2;
IA	Inflammatory Aneurysm;
UC-MSCs	Umbilical Cord Mesenchymal Stem Cells;
NF-κB	Nuclear Factor Kappa B;
AngII	Angiotensin II;
AT1R	Angiotensin II Type 1 Receptor;
NICD	Notch Intracellular Domain;
PI3K	Phosphoinositide 3-Kinase;
Akt	Protein Kinase B;
Wnt	Wnt Signaling Pathway;
ERK	Extracellular Signal-Regulated Kinase;
MAPK	Mitogen-Activated Protein Kinase;
JAK	Janus Kinase Janus;
STAT	Signal Transducer and Activator of Transcription;
ARBs	Angiotensin Receptor Blockers;
SUMO	Small Ubiquitin-like Modifier;
SENP1	SUMO-specific Protease 1;
SRF	Serum Response Factor;
ELK1	ETS-Like Gene 1;
ER	Estrogen Receptor;
GSDMD	Gasdermin D;
ODC1	Ornithine Decarboxylase 1;
CHOP	C/EBP Homologous Protein;
NRP1	Neuropilin-1;
ITGB3	Integrin Beta-3;
GLUT1	Glucose Transporter 1;
A7r5	Rat Aortic Smooth Muscle Cell Line;
TCA	Tricarboxylic Acid Cycle;
FAO	Fatty Acid Oxidation;
CPT1	Carnitine Palmitoyltransferase 1;
CPT2	Carnitine Palmitoyltransferase 2;
IL-1β	Interleukin-1 Beta;
IFN-γ	Interferon Gamma;
MCP-1	Monocyte Chemoattractant Protein-1;
FAS	Fatty Acid Synthase;
CD95	Cluster of Differentiation 95;
ZBP1	ZDNA Binding Protein 1;
AIM2	Absent in Melanoma 2;
TFEB	Transcription Factor EB;
2HPBCD	2-Hydroxypropyl-β-Cyclodextrin;
BCL-2	B-Cell Lymphoma 2;
SMOC2	SMAD Family Member 4 Interacting Protein 2;
JNK	c-Jun N-Terminal Kinase c-Jun;
SAPK	Stress-Activated Protein Kinase;
MAPK	Mitogen-Activated Protein Kinase;
iNOS	Inducible Nitric Oxide Synthase;
CD	Cluster of Differentiation;
Nox4	NADPH Oxidase 4;
BAF60α	Bromodomain Adjacent to Zinc Finger 60α;
DAMPs	Damage-Associated Molecular Patterns.

## References

[1] Davis F.M., Daugherty A., Lu H.S. (2019). Updates of recent aortic aneurysm research. Arterioscler. Thromb. Vasc. Biol..

[2] Altobelli E., Rapacchietta L., Profeta V.F., Fagnano R. (2018). Risk factors for abdominal aortic aneurysm in population-based studies: A systematic review and meta-analysis. Int. J. Environ. Res. Public Health.

[3] Johnston K.W., Rutherford R.B., Tilson M.D., Shah D.M., Hollier L., Stanley J.C. (1991). Suggested standards for reporting on arterial aneurysms. J. Vasc. Surg..

[4] Howard D., Banerjee A., Fairhead J., Handa A., Silver L., Rothwell P. (2015). Age-specific incidence, risk factors and outcome of acute abdominal aortic aneurysms in a defined population. J. Br. Surg..

[5] Golledge J., Krishna S.M., Wang Y. (2022). Mouse models for abdominal aortic aneurysm. Br. J. Pharmacol..

[6] Nordon I.M., Hinchliffe R.J., Loftus I.M., Thompson M.M. (2011). Pathophysiology and epidemiology of abdominal aortic aneurysms. Nat. Rev. Cardiol..

[7] Seven M. (2024). Abdominal Aortic Aneurysm. Master’s Thesis.

[8] Sampson U.K., Norman P.E., Fowkes F.G.R., Aboyans V., Song Y., Harrell F.E., Forouzanfar M.H., Naghavi M., Denenberg J.O., McDermott M.M. (2014). Estimation of global and regional incidence and prevalence of abdominal aortic aneurysms 1990 to 2010. Glob. Heart.

[9] Owens D.K., Davidson K.W., Krist A.H., Barry M.J., Cabana M., Caughey A.B., Doubeni C.A., Epling J.W., Kubik M., Landefeld C.S. (2019). Screening for abdominal aortic aneurysm: US preventive services task force recommendation statement. JAMA.

[10] Li K., Zhang K., Li T., Zhai S. (2018). Primary results of abdominal aortic aneurysm screening in the at-risk residents in middle China. BMC Cardiovasc. Disord..

[11] Song P., He Y., Adeloye D., Zhu Y., Ye X., Yi Q., Rahimi K., Rudan I., Group G.H.E.R. (2023). The global and regional prevalence of abdominal aortic aneurysms: A systematic review and modeling analysis. Ann. Surg..

[12] Lederle F.A., Kyriakides T.C., Stroupe K.T., Freischlag J.A., Padberg F.T., Matsumura J.S., Huo Z., Johnson G.R. (2019). Open versus endovascular repair of abdominal aortic aneurysm. N. Engl. J. Med..

[13] Paravastu S.C.V., Jayarajasingam R., Cottam R., Palfreyman S.J., Michaels J.A., Thomas S.M. (2014). Endovascular repair of abdominal aortic aneurysm. Cochrane Database Syst. Rev..

[14] Quintana R.A., Taylor W.R. (2019). Cellular mechanisms of aortic aneurysm formation. Circ. Res..

[15] Qian G., Adeyanju O., Olajuyin A., Guo X. (2022). Abdominal aortic aneurysm formation with a focus on vascular smooth muscle cells. Life.

[16] Wagenhäuser M.U., Mulorz J., Krott K.J., Bosbach A., Feige T., Rhee Y.H., Chatterjee M., Petzold N., Böddeker C., Ibing W. (2024). Crosstalk of platelets with macrophages and fibroblasts aggravates inflammation, aortic wall stiffening, and osteopontin release in abdominal aortic aneurysm. Cardiovasc. Res..

[17] Lu H., Du W., Ren L., Hamblin M.H., Becker R.C., Chen Y.E., Fan Y. (2021). Vascular smooth muscle cells in aortic aneurysm: From genetics to mechanisms. J. Am. Heart Assoc..

[18] Sinha S., Iyer D., Granata A. (2014). Embryonic origins of human vascular smooth muscle cells: Implications for in vitro modeling and clinical application. Cell. Mol. Life Sci..

[19] Amali A.A., Sie L., Winkler C., Featherstone M. (2013). Zebrafish hoxd4a acts upstream of meis1. 1 to direct vasculogenesis, angiogenesis and hematopoiesis. PLoS ONE.

[20] Carmeliet P. (2000). Mechanisms of angiogenesis and arteriogenesis. Nat. Med..

[21] Mironov A.A., Beznoussenko G.V. (2022). Opinion: On the way towards the new paradigm of atherosclerosis. Int. J. Mol. Sci..

[22] Pouget C., Pottin K., Jaffredo T. (2008). Sclerotomal origin of vascular smooth muscle cells and pericytes in the embryo. Dev. Biol..

[23] Gadson P.F., Dalton M.L., Patterson E., Svoboda D.D., Hutchinson L., Schram D., Rosenquist T.H. (1997). Differential response of mesoderm-and neural crest-derived smooth muscle to TGF-β1: Regulation of c-myb and α1 (I) procollagen genes. Exp. Cell Res..

[24] Pfaltzgraff E.R., Bader D.M. (2015). Heterogeneity in vascular smooth muscle cell embryonic origin in relation to adult structure, physiology, and disease. Dev. Dyn..

[25] Cheung C., Bernardo A.S., Trotter M.W., Pedersen R.A., Sinha S. (2012). Generation of human vascular smooth muscle subtypes provides insight into embryological origin–dependent disease susceptibility. Nat. Biotechnol..

[26] Majesky M.W. (2007). Developmental basis of vascular smooth muscle diversity. Arterioscler. Thromb. Vasc. Biol..

[27] Cho M.J., Lee M.-R., Park J.-G. (2023). Aortic aneurysms: Current pathogenesis and therapeutic targets. Exp. Mol. Med..

[28] Golledge J., Norman P.E. (2010). Atherosclerosis and Abdominal Aortic Aneurysm: Cause, Response, or Common Risk Factors?.

[29] Cao G., Xuan X., Hu J., Zhang R., Jin H., Dong H. (2022). How vascular smooth muscle cell phenotype switching contributes to vascular disease. Cell Commun. Signal..

[30] Diehm N., Dick F., Schaffner T., Schmidli J., Kalka C., Di Santo S., Voelzmann J., Baumgartner I. (2007). Novel insight into the pathobiology of abdominal aortic aneurysm and potential future treatment concepts. Prog. Cardiovasc. Dis..

[31] Halloran B.G., Davis V.A., McManus B.M., Lynch T.G., Baxter B.T. (1995). Localization of aortic disease is associated with intrinsic differences in aortic structure. J. Surg. Res..

[32] Tanweer O., Wilson T.A., Metaxa E., Riina H.A., Meng H. (2014). A comparative review of the hemodynamics and pathogenesis of cerebral and abdominal aortic aneurysms: Lessons to learn from each other. J. Cerebrovasc. Endovasc. Neurosurg..

[33] Dua M.M., Dalman R.L. (2010). Hemodynamic influences on abdominal aortic aneurysm disease: Application of biomechanics to aneurysm pathophysiology. Vasc. Pharmacol..

[34] Taylor C.A., Hughes T.J., Zarins C.K. (1999). Effect of exercise on hemodynamic conditions in the abdominal aorta. J. Vasc. Surg..

[35] Wolinsky H., Glagov S. (1967). A lamellar unit of aortic medial structure and function in mammals. Circ. Res..

[36] Zengin E., Chalajour F., Gehling U.M., Ito W.D., Treede H., Lauke H., Weil J., Reichenspurner H., Kilic N., Ergün S.l. (2006). Vascular wall resident progenitor cells: A source for postnatal vasculogenesis. Development.

[37] Torsney E., Xu Q. (2011). Resident vascular progenitor cells. J. Mol. Cell. Cardiol..

[38] Majesky M.W., Horita H., Ostriker A., Lu S., Regan J.N., Bagchi A., Dong X.R., Poczobutt J., Nemenoff R.A., Weiser-Evans M.C. (2017). Differentiated smooth muscle cells generate a subpopulation of resident vascular progenitor cells in the adventitia regulated by Klf4. Circ. Res..

[39] Psaltis P.J., Simari R.D. (2015). Vascular wall progenitor cells in health and disease. Circ. Res..

[40] Crosas-Molist E., Meirelles T., López-Luque J., Serra-Peinado C., Selva J., Caja L., Gorbenko del Blanco D., Uriarte J.J., Bertran E., Mendizábal Y. (2015). Vascular smooth muscle cell phenotypic changes in patients with Marfan syndrome. Arterioscler. Thromb. Vasc. Biol..

[41] Chen Q., Zhang H., Liu Y., Adams S., Eilken H., Stehling M., Corada M., Dejana E., Zhou B., Adams R.H. (2016). Endothelial cells are progenitors of cardiac pericytes and vascular smooth muscle cells. Nat. Commun..

[42] Galkina E., Ley K. (2009). Immune and inflammatory mechanisms of atherosclerosis. Annu. Rev. Immunol..

[43] Hansson G.K., Hermansson A. (2011). The immune system in atherosclerosis. Nat. Immunol..

[44] Moos M.P., John N., Gräbner R., Noßmann S., Günther B., Vollandt R.d., Funk C.D., Kaiser B., Habenicht A.J. (2005). The lamina adventitia is the major site of immune cell accumulation in standard chow-fed apolipoprotein E–deficient mice. Arterioscler. Thromb. Vasc. Biol..

[45] Basatemur G.L., Jørgensen H.F., Clarke M.C., Bennett M.R., Mallat Z. (2019). Vascular smooth muscle cells in atherosclerosis. Nat. Rev. Cardiol..

[46] Caplice N.M., Doyle B. (2005). Vascular progenitor cells: Origin and mechanisms of mobilization, differentiation, integration, and vasculogenesis. Stem Cells Dev..

[47] Pohl U. (2020). Connexins: Key players in the control of vascular plasticity and function. Physiol. Rev..

[48] Cowan D.B., Lye S.J., Langille B.L. (1998). Regulation of vascular connexin43 gene expression by mechanical loads. Circ. Res..

[49] Berrier A.L., Yamada K.M. (2007). Cell–matrix adhesion. J. Cell. Physiol..

[50] Liu J., Zhang J., Fu X., Yang S., Li Y., Liu J., DiSanto M.E., Chen P., Zhang X. (2023). The emerging role of cell adhesion molecules on benign prostatic hyperplasia. Int. J. Mol. Sci..

[51] Mui K.L., Bae Y.H., Gao L., Liu S.-L., Xu T., Radice G.L., Chen C.S., Assoian R.K. (2015). N-cadherin induction by ECM stiffness and FAK overrides the spreading requirement for proliferation of vascular smooth muscle cells. Cell Rep..

[52] Feng X., Li X., Yang C., Ren Q., Zhang W., Li N., Zhang M., Zhang B., Zhang L., Zhou X. (2019). Maternal High-Sucrose Diet Accelerates Vascular Stiffness in Aged Offspring via Suppressing Cav1. 2 and Contractile Phenotype of Vascular Smooth Muscle Cells. Mol. Nutr. Food Res..

[53] Huang H., Sun Z., Hill M.A., Meininger G.A. (2018). A calcium mediated mechanism coordinating vascular smooth muscle cell adhesion during kcl activation. Front. Physiol..

[54] Somlyo A.P., Somlyo A.V. (2003). Ca2+ sensitivity of smooth muscle and nonmuscle myosin II: Modulated by G proteins, kinases, and myosin phosphatase. Physiol. Rev..

[55] Bogunovic N., Meekel J.P., Micha D., Blankensteijn J.D., Hordijk P.L., Yeung K.K. (2019). Impaired smooth muscle cell contractility as a novel concept of abdominal aortic aneurysm pathophysiology. Sci. Rep..

[56] Au D.T., Ying Z., Hernández-Ochoa E.O., Fondrie W.E., Hampton B., Migliorini M., Galisteo R., Schneider M.F., Daugherty A., Rateri D.L. (2018). LRP1 (low-density lipoprotein receptor–related protein 1) regulates smooth muscle contractility by modulating Ca2+ signaling and expression of cytoskeleton-related proteins. Arterioscler. Thromb. Vasc. Biol..

[57] Tomida S., Ishima T., Nagai R., Aizawa K. (2024). T-Type Voltage-Gated Calcium Channels: Potential Regulators of Smooth Muscle Contractility. Int. J. Mol. Sci..

[58] House S.J., Potier M., Bisaillon J., Singer H.A., Trebak M. (2008). The non-excitable smooth muscle: Calcium signaling and phenotypic switching during vascular disease. Pflügers Arch.-Eur. J. Physiol..

[59] Li W., Zhang Y., Yang F., Zhang L. (2024). Molecular Mechanism by Which TRPC6 Regulates Calcium Signaling and Neuroinflammation in the Onset and Development of Ischemic Stroke: A Review. Altern. Ther. Health Med..

[60] Yin Q., Zang G., Li N., Sun C., Du R. (2022). Agonist-induced Piezo1 activation promote mitochondrial-dependent apoptosis in vascular smooth muscle cells. BMC Cardiovasc. Disord..

[61] Tsai M.-C., Chen L., Zhou J., Tang Z., Hsu T.-F., Wang Y., Shih Y.-T., Peng H.-H., Wang N., Guan Y. (2009). Shear stress induces synthetic-to-contractile phenotypic modulation in smooth muscle cells via peroxisome proliferator-activated receptor α/δ activations by prostacyclin released by sheared endothelial cells. Circ. Res..

[62] Jia L., Wang L., Wei F., Li C., Wang Z., Yu H., Chen H., Wang B., Jiang A. (2020). Effects of Caveolin-1-ERK1/2 pathway on endothelial cells and smooth muscle cells under shear stress. Exp. Biol. Med..

[63] Qiao M., Li Y., Yan S., Zhang R.J., Dong H. (2025). Modulation of arterial wall remodeling by mechanical stress: Focus on abdominal aortic aneurysm. Vasc. Med..

[64] Jeremy J.Y., Rowe D., Emsley A.M., Newby A.C. (1999). Nitric oxide and the proliferation of vascular smooth muscle cells. Cardiovasc. Res..

[65] Fu H., Shen Q.-R., Zhao Y., Ni M., Zhou C.-C., Chen J.-K., Chi C., Li D.-J., Liang G., Shen F.-M. (2022). Activating α7nAChR ameliorates abdominal aortic aneurysm through inhibiting pyroptosis mediated by NLRP3 inflammasome. Acta Pharmacol. Sin..

[66] Chatzizisis Y.S., Coskun A.U., Jonas M., Edelman E.R., Stone P.H., Feldman C.L. (2007). Risk stratification of individual coronary lesions using local endothelial shear stress: A new paradigm for managing coronary artery disease. Curr. Opin. Cardiol..

[67] Davis F.M., Tsoi L.C., Ma F., Wasikowski R., Moore B.B., Kunkel S.L., Gudjonsson J.E., Gallagher K.A. (2022). Single-cell transcriptomics reveals dynamic role of smooth muscle cells and enrichment of immune cell subsets in human abdominal aortic aneurysms. Ann. Surg..

[68] Qin H.-L., Bao J.-H., Tang J.-J., Xu D.-Y., Shen L. (2023). Arterial remodeling: The role of mitochondrial metabolism in vascular smooth muscle cells. Am. J. Physiol.-Cell Physiol..

[69] Liu M.-Y., Jin J., Li S.-L., Yan J., Zhen C.-L., Gao J.-L., Zhang Y.-H., Zhang Y.-Q., Shen X., Zhang L.-S. (2016). Mitochondrial fission of smooth muscle cells is involved in artery constriction. Hypertension.

[70] Mizrak D., Feng H., Yang B. (2022). Dissecting the heterogeneity of human thoracic aortic aneurysms using single-cell transcriptomics. Arterioscler. Thromb. Vasc. Biol..

[71] Lahiri V., Klionsky D.J. (2017). PHB2/prohibitin 2: An inner membrane mitophagy receptor. Cell Res..

[72] Tavris B.S., Peters A.S., Böckler D., Dihlmann S. (2023). Mitochondrial Dysfunction and Increased DNA Damage in Vascular Smooth Muscle Cells of Abdominal Aortic Aneurysm (AAA-SMC). Oxidative Med. Cell. Longev..

[73] Jia Y., Mao C., Ma Z., Huang J., Li W., Ma X., Zhang S., Li M., Yu F., Sun Y. (2022). PHB2 maintains the contractile phenotype of VSMCs by counteracting PKM2 splicing. Circ. Res..

[74] Wang D., Jia L., Zhao C., Wang H., Dai Z., Jing Y., Jiang B., Xin S. (2023). Mitochondrial quality control in abdominal aortic aneurysm: From molecular mechanisms to therapeutic strategies. FASEB J..

[75] Yuan Z., Lu Y., Wei J., Wu J., Yang J., Cai Z. (2021). Abdominal aortic aneurysm: Roles of inflammatory cells. Front. Immunol..

[76] Wortmann M., Skorubskaya E., Peters A.S., Hakimi M., Böckler D., Dihlmann S. (2019). Necrotic cell debris induces a NF-κB-driven inflammasome response in vascular smooth muscle cells derived from abdominal aortic aneurysms (AAA-SMC). Biochem. Biophys. Res. Commun..

[77] Qu R., Qu W. (2019). Metformin inhibits LPS-induced inflammatory response in VSMCs by regulating TLR4 and PPAR-γ. Eur. Rev. Med. Pharmacol. Sci..

[78] Wen H., Wang M., Gong S., Li X., Meng J., Wen J., Wang Y., Zhang S., Xin S. (2020). Human umbilical cord mesenchymal stem cells attenuate abdominal aortic aneurysm progression in sprague-dawley rats: Implication of vascular smooth muscle cell phenotypic modulation. Stem Cells Dev..

[79] Mi T., Nie B., Zhang C., Zhou H. (2011). The elevated expression of osteopontin and NF-κB in human aortic aneurysms and its implication. J. Huazhong Univ. Sci. Technol. Med. Sci..

[80] Han M.S., Jung D.Y., Morel C., Lakhani S.A., Kim J.K., Flavell R.A., Davis R.J. (2013). JNK expression by macrophages promotes obesity-induced insulin resistance and inflammation. Science.

[81] Magnani F., Mattevi A. (2019). Structure and mechanisms of ROS generation by NADPH oxidases. Curr. Opin. Struct. Biol..

[82] Xiong W., Mactaggart J., Knispel R., Worth J., Zhu Z., Li Y., Sun Y., Baxter B.T., Johanning J. (2009). Inhibition of reactive oxygen species attenuates aneurysm formation in a murine model. Atherosclerosis.

[83] He F., Zuo L. (2015). Redox roles of reactive oxygen species in cardiovascular diseases. Int. J. Mol. Sci..

[84] Krylatov A.V., Maslov L.N., Voronkov N.S., Boshchenko A.A., Popov S.V., Gomez L., Wang H., Jaggi A.S., Downey J.M. (2018). Reactive oxygen species as intracellular signaling molecules in the cardiovascular system. Curr. Cardiol. Rev..

[85] Ren J., Han Y., Ren T., Fang H., Xu X., Lun Y., Jiang H., Xin S., Zhang J. (2020). AEBP1 promotes the occurrence and development of abdominal aortic aneurysm by modulating inflammation via the NF-κB pathway. J. Atheroscler. Thromb..

[86] Zhong L., He X., Si X., Wang H., Li B., Hu Y., Li M., Chen X., Liao W., Liao Y. (2019). SM22α (smooth muscle 22α) prevents aortic aneurysm formation by inhibiting smooth muscle cell phenotypic switching through suppressing reactive oxygen species/NF-κB (nuclear factor-κB). Arterioscler. Thromb. Vasc. Biol..

[87] Sanchez-Infantes D., Nus M., Navas-Madronal M., Fite J., Perez B., Barros-Membrilla A.J., Soto B., Martinez-Gonzalez J., Camacho M., Rodriguez C. (2021). Oxidative Stress and Inflammatory Markers in Abdominal Aortic Aneurysm. Antioxidants.

[88] Tsai S.H., Hsu L.A., Tsai H.Y., Yeh Y.H., Lu C.Y., Chen P.C., Wang J.C., Chiu Y.L., Lin C.Y., Hsu Y.J. (2020). Aldehyde dehydrogenase 2 protects against abdominal aortic aneurysm formation by reducing reactive oxygen species, vascular inflammation, and apoptosis of vascular smooth muscle cells. FASEB J..

[89] Shi J., Yang Y., Cheng A., Xu G., He F. (2020). Metabolism of vascular smooth muscle cells in vascular diseases. Am. J. Physiol.-Heart Circ. Physiol..

[90] Chung J., Lachapelle K., Wener E., Cartier R., De Varennes B., Fraser R., Leask R.L. (2014). Energy loss, a novel biomechanical parameter, correlates with aortic aneurysm size and histopathologic findings. J. Thorac. Cardiovasc. Surg..

[91] Kuznetsov A.V., Hermann M., Saks V., Hengster P., Margreiter R. (2009). The cell-type specificity of mitochondrial dynamics. Int. J. Biochem. Cell Biol..

[92] Marchi S., Guilbaud E., Tait S.W., Yamazaki T., Galluzzi L. (2023). Mitochondrial control of inflammation. Nat. Rev. Immunol..

[93] Xia D., Chen Y., Luo G., Wei D. (2023). Atherosclerosis: From the Disruption of Mitochondrial Membrane Potential to the Potential Interventional Strategies. Curr. Med. Chem..

[94] Paul W., Hruz M.M.M. (2001). Structural analysis of the GLUT1 facilitative glucose transporter. Mol. Membr. Biol..

[95] Hall J.L., Chatham J.C., Eldar-Finkelman H., Gibbons G.H. (2001). Upregulation of glucose metabolism during intimal lesion formation is coupled to the inhibition of vascular smooth muscle cell apoptosis: Role of GSK3β. Diabetes.

[96] Lin C.-Y., Hsu S.-C., Lee H.-S., Lin S.-H., Tsai C.-S., Huang S.-M., Shih C.-C., Hsu Y.-J. (2013). Enhanced expression of glucose transporter-1 in vascular smooth muscle cells via the Akt/tuberous sclerosis complex subunit 2 (TSC2)/mammalian target of rapamycin (mTOR)/ribosomal S6 protein kinase (S6K) pathway in experimental renal failure. J. Vasc. Surg..

[97] Zhou Q., Xu J., Liu M., He L., Zhang K., Yang Y., Yang X., Zhou H., Tang M., Lu L. (2019). Warburg effect is involved in apelin-13-induced human aortic vascular smooth muscle cells proliferation. J. Cell. Physiol..

[98] Pfleger J., He M., Abdellatif M. (2015). Mitochondrial complex II is a source of the reserve respiratory capacity that is regulated by metabolic sensors and promotes cell survival. Cell Death Dis..

[99] Perez J., Hill B.G., Benavides G.A., Dranka B.P., Darley-Usmar V.M. (2010). Role of cellular bioenergetics in smooth muscle cell proliferation induced by platelet-derived growth factor. Biochem. J..

[100] Salabei J.K., Hill B.G. (2013). Mitochondrial fission induced by platelet-derived growth factor regulates vascular smooth muscle cell bioenergetics and cell proliferation. Redox Biol..

[101] Chen X., Austin E.D., Talati M., Fessel J.P., Farber-Eger E.H., Brittain E.L., Hemnes A.R., Loyd J.E., West J. (2017). Oestrogen inhibition reverses pulmonary arterial hypertension and associated metabolic defects. Eur. Respir. J..

[102] Barron J.T., Kopp S.J., Tow J., Parrillo J.E. (1994). Fatty acid, tricarboxylic acid cycle metabolites, and energy metabolism in vascular smooth muscle. Am. J. Physiol.-Heart Circ. Physiol..

[103] Scheede-Bergdahl C., Bergdahl A. (2017). Adaptation of mitochondrial expression and ATP production in dedifferentiating vascular smooth muscle cells. Can. J. Physiol. Pharmacol..

[104] Li Y., Du Y., Zhou Y., Chen Q., Luo Z., Ren Y., Chen X., Chen G. (2023). Iron and copper: Critical executioners of ferroptosis, cuproptosis and other forms of cell death. Cell Commun. Signal..

[105] Cui H., Chen Y., Li K., Zhan R., Zhao M., Xu Y., Lin Z., Fu Y., He Q., Tang P.C. (2021). Untargeted metabolomics identifies succinate as a biomarker and therapeutic target in aortic aneurysm and dissection. Eur. Heart J..

[106] Wu H., Li Z., Yang L., He L., Liu H., Yang S., Xu Q., Li Y., Li W., Li Y. (2024). ANK Deficiency-Mediated Cytosolic Citrate Accumulation Promotes Aortic Aneurysm. Circ. Res..

[107] Sun L.-Y., Lyu Y.-Y., Zhang H.-Y., Shen Z., Lin G.-Q., Geng N., Wang Y.-L., Huang L., Feng Z.-H., Guo X. (2022). Nuclear receptor NR1D1 regulates abdominal aortic aneurysm development by targeting the mitochondrial tricarboxylic acid cycle enzyme aconitase-2. Circulation.

[108] Gao J., Chen Y., Wang H., Li X., Li K., Xu Y., Xie X., Guo Y., Yang N., Zhang X. (2023). Gasdermin D deficiency in vascular smooth muscle cells ameliorates abdominal aortic aneurysm through reducing putrescine synthesis. Adv. Sci..

[109] Lai C.-H., Chang C.-W., Lee F.-T., Kuo C.-H., Hsu J.-H., Liu C.-P., Wu H.-L., Yeh J.-L. (2020). Targeting vascular smooth muscle cell dysfunction with xanthine derivative KMUP-3 inhibits abdominal aortic aneurysm in mice. Atherosclerosis.

[110] Song T., Zhao S., Luo S., Chen C., Liu X., Wu X., Sun Z., Cao J., Wang Z., Wang Y. (2024). SLC44A2 regulates vascular smooth muscle cell phenotypic switching and aortic aneurysm. J. Clin. Investig..

[111] Owens G.K., Kumar M.S., Wamhoff B.R. (2004). Molecular regulation of vascular smooth muscle cell differentiation in development and disease. Physiol. Rev..

[112] Chistiakov D.A., Orekhov A.N., Bobryshev Y.V. (2015). Vascular smooth muscle cell in atherosclerosis. Acta Physiol..

[113] Raines E.W. (2004). PDGF and cardiovascular disease. Cytokine Growth Factor Rev..

[114] Zaidi M., Lizneva D., Yuen T. (2021). The role of PDGF-BB in the bone-vascular relationship during aging. J. Clin. Investig..

[115] Zhou J., Shao L., Yu J., Huang J., Feng Q. (2021). PDGF-BB promotes vascular smooth muscle cell migration by enhancing Pim-1 expression via inhibiting miR-214. Ann. Transl. Med..

[116] Tian J., Fu Y., Li Q., Xu Y., Xi X., Zheng Y., Yu L., Wang Z., Yu B., Tian J. (2020). Differential expression and bioinformatics analysis of CircRNA in PDGF-BB-induced vascular smooth muscle cells. Front. Genet..

[117] Han J.-H., Park H.-S., Lee D.-H., Jo J.-H., Heo K.-S., Myung C.-S. (2021). Regulation of autophagy by controlling Erk1/2 and mTOR for platelet-derived growth factor-BB-mediated vascular smooth muscle cell phenotype shift. Life Sci..

[118] Swaminathan G., Stoilov I., Broekelmann T., Mecham R., Ramamurthi A. (2018). Phenotype-based selection of bone marrow mesenchymal stem cell-derived smooth muscle cells for elastic matrix regenerative repair in abdominal aortic aneurysms. J. Tissue Eng. Regen. Med..

[119] Chen J., Cui X., Qian Z., Li Y., Kang K., Qu J., Li L., Gou D. (2016). Multi-omics analysis reveals regulators of the response to PDGF-BB treatment in pulmonary artery smooth muscle cells. BMC Genom..

[120] Song X., Shi J., Liu J., Liu Y., Yu Y., Qiu Y., Cao Z., Pan Y., Yuan X., Chu Y. (2022). Recombinant truncated latency-associated peptide alleviates liver fibrosis in vitro and in vivo via inhibition of TGF-β/Smad pathway. Mol. Med..

[121] Ghosh J., Murphy M.O., Turner N., Khwaja N., Halka A., Kielty C.M., Walker M.G. (2005). The role of transforming growth factor β1 in the vascular system. Cardiovasc. Pathol..

[122] Bobik A. (2006). Transforming growth factor-βs and vascular disorders. Arterioscler. Thromb. Vasc. Biol..

[123] Derynck R., Zhang Y.E. (2003). Smad-dependent and Smad-independent pathways in TGF-β family signalling. Nature.

[124] Gillis E., Van Laer L., Loeys B.L. (2013). Genetics of thoracic aortic aneurysm: At the crossroad of transforming growth factor-β signaling and vascular smooth muscle cell contractility. Circ. Res..

[125] Milewicz D.M., Guo D.-C., Tran-Fadulu V., Lafont A.L., Papke C.L., Inamoto S., Kwartler C.S., Pannu H. (2008). Genetic basis of thoracic aortic aneurysms and dissections: Focus on smooth muscle cell contractile dysfunction. Annu. Rev. Genom. Hum. Genet..

[126] Qi Y., Liang X., Dai F., Guan H., Sun J., Yao W. (2020). RhoA/ROCK pathway activation is regulated by AT1 receptor and participates in smooth muscle migration and dedifferentiation via promoting actin cytoskeleton polymerization. Int. J. Mol. Sci..

[127] Belmadani S., Zerfaoui M., Boulares H.A., Palen D.I., Matrougui K. (2008). Microvessel vascular smooth muscle cells contribute to collagen type I deposition through ERK1/2 MAP kinase, αvβ3-integrin, and TGF-β1 in response to ANG II and high glucose. Am. J. Physiol.-Heart Circ. Physiol..

[128] Da X., Li Z., Huang X., He Z., Yu Y., Tian T., Xu C., Yao Y., Wang Q.K. (2023). AGGF1 therapy inhibits thoracic aortic aneurysms by enhancing integrin α7-mediated inhibition of TGF-β1 maturation and ERK1/2 signaling. Nat. Commun..

[129] Dai X., Shen J., Priyanka Annam N., Jiang H., Levi E., Schworer C.M., Tromp G., Arora A., Higgins M., Wang X.-F. (2015). SMAD3 deficiency promotes vessel wall remodeling, collagen fiber reorganization and leukocyte infiltration in an inflammatory abdominal aortic aneurysm mouse model. Sci. Rep..

[130] Shi Y., Massagué J. (2003). Mechanisms of TGF-β signaling from cell membrane to the nucleus. Cell.

[131] Rombouts K.B., van Merrienboer T.A., Ket J.C., Bogunovic N., van der Velden J., Yeung K.K. (2022). The role of vascular smooth muscle cells in the development of aortic aneurysms and dissections. Eur. J. Clin. Investig..

[132] Elmarasi M., Elmakaty I., Elsayed B., Elsayed A., Zein J.A., Boudaka A., Eid A.H. (2024). Phenotypic switching of vascular smooth muscle cells in atherosclerosis, hypertension, and aortic dissection. J. Cell. Physiol..

[133] He X., Li X., Han Y., Chen G., Xu T., Cai D., Sun Y., Wang S., Lai Y., Teng Z. (2022). CircRNA Chordc1 protects mice from abdominal aortic aneurysm by contributing to the phenotype and growth of vascular smooth muscle cells. Mol. Ther.-Nucleic Acids.

[134] Lin F., Yang X. (2010). TGF-β signaling in aortic aneurysm: Another round of controversy. J. Genet. Genom..

[135] Meester J.A., Vandeweyer G., Pintelon I., Lammens M., Van Hoorick L., De Belder S., Waitzman K., Young L., Markham L.W., Vogt J. (2017). Loss-of-function mutations in the X-linked biglycan gene cause a severe syndromic form of thoracic aortic aneurysms and dissections. Genet. Med..

[136] Yorn C., Kim H., Jeong K. (2024). Influence of DNA methylation on vascular smooth muscle cell phenotypic switching. Int. J. Mol. Sci..

[137] Tingting T., Wenjing F., Qian Z., Hengquan W., Simin Z., Zhisheng J., Shunlin Q. (2020). The TGF-β pathway plays a key role in aortic aneurysms. Clin. Chim. Acta.

[138] Shi Y., Liu L., Gong Y., Zhang C., Yang Y., Wang W., Qin L. (2025). Isovaleroylbinankadsurin A ameliorates atherosclerosis and restenosis by promoting LXRα signaling pathway and inhibiting TGF-β1 and FHL1 signaling pathway. Phytomedicine.

[139] High F.A., Zhang M., Proweller A., Tu L., Parmacek M.S., Pear W.S., Epstein J.A. (2007). An essential role for Notch in neural crest during cardiovascular development and smooth muscle differentiation. J. Clin. Investig..

[140] Boucher J., Gridley T., Liaw L. (2012). Molecular pathways of notch signaling in vascular smooth muscle cells. Front. Physiol..

[141] Sharma N., Dev R., Ruiz-Rosado J.d.D., Partida-Sanchez S., Guerau-de-Arellano M., Dhakal P., Kuivaniemi H., Hans C.P. (2019). Pharmacological inhibition of Notch signaling regresses pre-established abdominal aortic aneurysm. Sci. Rep..

[142] Hans C.P., Sharma N., Dev R., Blain J.M., Tonniges J., Agarwal G. (2020). DAPT, a potent Notch inhibitor regresses actively growing abdominal aortic aneurysm via divergent pathways. Clin. Sci..

[143] Zheng Y.-H., Li F.-D., Tian C., Ren H.-L., Du J., Li H.-H. (2013). Notch γ-secretase inhibitor dibenzazepine attenuates angiotensin II-induced abdominal aortic aneurysm in ApoE knockout mice by multiple mechanisms. PLoS ONE.

[144] Breikaa R. (2022). Investigating the Role of Endothelial Cell-Expressed Jag1 on Smooth Muscle Function and Vascular Homeostasis. Ph.D. Thesis.

[145] Xie M., Li X., Chen L., Zhang Y., Chen L., Hua H., Qi J. (2024). The crosstalks between vascular endothelial cells, vascular smooth muscle cells, and adventitial fibroblasts in vascular remodeling. Life Sci..

[146] Ozasa Y., Akazawa H., Qin Y., Tateno K., Ito K., Kudo-Sakamoto Y., Yano M., Yabumoto C., Naito A.T., Oka T. (2013). Notch activation mediates angiotensin II-induced vascular remodeling by promoting the proliferation and migration of vascular smooth muscle cells. Hypertens. Res..

[147] Pan L., Gross K.W. (2005). Transcriptional regulation of renin: An update. Hypertension.

[148] Tang Y., Urs S., Liaw L. (2008). Hairy-related transcription factors inhibit notch-induced smooth muscle α-actin expression by interfering with notch intracellular Domain/CBF-1 complex interaction with the CBF-1–binding site. Circ. Res..

[149] Boulos N., Helle F., Dussaule J.-C., Placier S., Milliez P., Djudjaj S., Guerrot D., Joutel A., Ronco P., Boffa J.-J. (2011). Notch3 is essential for regulation of the renal vascular tone. Hypertension.

[150] Kuba K., Imai Y., Penninger J.M. (2013). Multiple functions of angiotensin-converting enzyme 2 and its relevance in cardiovascular diseases. Circ. J..

[151] Tan W.S.D., Liao W., Zhou S., Mei D., Wong W.-S.F. (2018). Targeting the renin–angiotensin system as novel therapeutic strategy for pulmonary diseases. Curr. Opin. Pharmacol..

[152] Kuba K., Imai Y., Ohto-Nakanishi T., Penninger J.M. (2010). Trilogy of ACE2: A peptidase in the renin–angiotensin system, a SARS receptor, and a partner for amino acid transporters. Pharmacol. Ther..

[153] Perlot T., Penninger J.M. (2013). ACE2–From the renin–angiotensin system to gut microbiota and malnutrition. Microbes Infect..

[154] Osumi H., Matsusaka S., Wakatsuki T., Suenaga M., Shinozaki E., Mizunuma N. (2015). Angiotensin II type-1 receptor blockers enhance the effects of bevacizumab-based chemotherapy in metastatic colorectal cancer patients. Mol. Clin. Oncol..

[155] Bosnyak S., Jones E.S., Christopoulos A., Aguilar M.-I., Thomas W.G., Widdop R.E. (2011). Relative affinity of angiotensin peptides and novel ligands at AT1 and AT2 receptors. Clin. Sci..

[156] Imai Y., Kuba K., Rao S., Huan Y., Guo F., Guan B., Yang P., Sarao R., Wada T., Leong-Poi H. (2005). Angiotensin-converting enzyme 2 protects from severe acute lung failure. Nature.

[157] Matysiak-Burzyńska Z.E., Nowakowska M., Domińska K., Kowalska K., Płuciennik E., Piastowska-Ciesielska A.W. (2018). Silencing of angiotensin receptor 1 interferes with angiotensin II oncogenic activity in endometrial cancer. J. Cell. Biochem..

[158] Speck D., Kleinau G., Szczepek M., Kwiatkowski D., Catar R., Philippe A., Scheerer P. (2022). Angiotensin and endothelin receptor structures with implications for signaling regulation and pharmacological targeting. Front. Endocrinol..

[159] Daugherty A., Cassis L. (1999). Chronic angiotensin II infusion promotes atherogenesis in low density lipoprotein receptor−/− mice. Ann. N. Y. Acad. Sci..

[160] Dong C.X., Malecki C., Robertson E., Hambly B., Jeremy R. (2023). Molecular mechanisms in genetic aortopathy–signaling pathways and potential interventions. Int. J. Mol. Sci..

[161] Norambuena-Soto I., Ocaranza M.P., Cancino-Arenas N., Sanhueza–Olivares F., Villar-Fincheira P., Leiva–Navarrete S., Mancilla-Medina C., Moya J., Novoa U., Jalil J.E. (2020). Angiotensin-(1–9) prevents vascular remodeling by decreasing vascular smooth muscle cell dedifferentiation through a FoxO1-dependent mechanism. Biochem. Pharmacol..

[162] Savoia C., Burger D., Nishigaki N., Montezano A., Touyz R.M. (2011). Angiotensin II and the vascular phenotype in hypertension. Expert Rev. Mol. Med..

[163] Ma J., Li Y., Yang X., Liu K., Zhang X., Zuo X., Ye R., Wang Z., Shi R., Meng Q. (2023). Signaling pathways in vascular function and hypertension: Molecular mechanisms and therapeutic interventions. Signal Transduct. Target. Ther..

[164] Karasaki K., Kokubo H., Bumdelger B., Kaji N., Sakai C., Ishida M., Yoshizumi M. (2023). Angiotensin II Type 1 Receptor Blocker Prevents Abdominal Aortic Aneurysm Progression in Osteoprotegerin-Deficient Mice via Upregulation of Angiotensin (1–7). J. Am. Heart Assoc..

[165] Hackam D.G., Thiruchelvam D., Redelmeier D.A. (2006). Angiotensin-converting enzyme inhibitors and aortic rupture: A population-based case-control study. Lancet.

[166] Bicknell C.D., Kiru G., Falaschetti E., Powell J.T., Poulter N.R., Collaborators A., Collaborators A. (2016). An evaluation of the effect of an angiotensin-converting enzyme inhibitor on the growth rate of small abdominal aortic aneurysms: A randomized placebo-controlled trial (AARDVARK). Eur. Heart J..

[167] Kristensen K.E., Torp-Pedersen C., Gislason G.H., Egfjord M., Rasmussen H.B., Hansen P.R. (2015). Angiotensin-converting enzyme inhibitors and angiotensin II receptor blockers in patients with abdominal aortic aneurysms: Nation-wide cohort study. Arterioscler. Thromb. Vasc. Biol..

[168] Sweeting M.J., Thompson S.G., Brown L.C., Greenhalgh R.M., Powell J.T. (2010). Use of angiotensin converting enzyme inhibitors is associated with increased growth rate of abdominal aortic aneurysms. J. Vasc. Surg..

[169] Tian K., Thanigaimani S., Gibson K., Golledge J. (2024). Systematic Review Examining the Association Between Angiotensin Converting Enzyme Inhibitor or Angiotensin Receptor Blocker Prescription and Abdominal Aortic Aneurysm Growth and Events. Eur. J. Vasc. Endovasc. Surg..

[170] Iida Y., Xu B., Schultz G.M., Chow V., White J.J., Sulaimon S., Hezi-Yamit A., Peterson S.R., Dalman R.L. (2012). Efficacy and mechanism of angiotensin II receptor blocker treatment in experimental abdominal aortic aneurysms. PLoS ONE.

[171] Kaschina E., Schrader F., Sommerfeld M., Kemnitz U.R., Grzesiak A., Krikov M., Unger T. (2008). Telmisartan prevents aneurysm progression in the rat by inhibiting proteolysis, apoptosis and inflammation. J. Hypertens..

[172] Krueger F., Kappert K., Foryst-Ludwig A., Kramer F., Clemenz M., Grzesiak A., Sommerfeld M., Paul Frese J., Greiner A., Kintscher U. (2017). AT1-receptor blockade attenuates outward aortic remodeling associated with diet-induced obesity in mice. Clin. Sci..

[173] Sakaue T., Suzuki J., Hamaguchi M., Suehiro C., Tanino A., Nagao T., Uetani T., Aono J., Nakaoka H., Kurata M. (2017). Perivascular adipose tissue angiotensin II type 1 receptor promotes vascular inflammation and aneurysm formation. Hypertension.

[174] Xuan H., Xu B., Wang W., Tanaka H., Fujimura N., Miyata M., Michie S.A., Dalman R.L. (2018). Inhibition or deletion of angiotensin II type 1 receptor suppresses elastase-induced experimental abdominal aortic aneurysms. J. Vasc. Surg..

[175] Tanaka H., Sukhova G., Schwartz D., Libby P. (1996). Proliferating arterial smooth muscle cells after balloon injury express TNF-α but not interleukin-1 or basic fibroblast growth factor. Arterioscler. Thromb. Vasc. Biol..

[176] Chou C.-C., Wang C.-P., Chen J.-H., Lin H.-H. (2019). Anti-atherosclerotic effect of Hibiscus leaf polyphenols against tumor necrosis factor-alpha-induced abnormal vascular smooth muscle cell migration and proliferation. Antioxidants.

[177] Fan W., Liu Y., Li C., Qu X., Zheng G., Zhang Q., Pan Z., Wang Y., Rong J. (2020). microRNA-331-3p maintains the contractile type of vascular smooth muscle cells by regulating TNF-α and CD14 in intracranial aneurysm. Neuropharmacology.

[178] Zhuang J., Luan P., Li H., Wang K., Zhang P., Xu Y., Peng W. (2017). The Yin–Yang dynamics of DNA methylation is the key regulator for smooth muscle cell phenotype switch and vascular remodeling. Arterioscler. Thromb. Vasc. Biol..

[179] Satta R., Maloku E., Zhubi A., Pibiri F., Hajos M., Costa E., Guidotti A. (2008). Nicotine decreases DNA methyltransferase 1 expression and glutamic acid decarboxylase 67 promoter methylation in GABAergic interneurons. Proc. Natl. Acad. Sci. USA.

[180] Dai Y., Chen D., Xu T. (2022). DNA methylation aberrant in atherosclerosis. Front. Pharmacol..

[181] Warsi A.A., Davies B., Morris-Stiff G., Hullin D., Lewis M.H. (2004). Abdominal aortic aneurysm and its correlation to plasma homocysteine, and vitamins. Eur. J. Vasc. Endovasc. Surg..

[182] Krishna S.M., Dear A., Craig J.M., Norman P.E., Golledge J. (2013). The potential role of homocysteine mediated DNA methylation and associated epigenetic changes in abdominal aortic aneurysm formation. Atherosclerosis.

[183] Lipp C., Lohoefer F., Reeps C., Rudelius M., Baummann M., Heemann U., Eckstein H.-H., Pelisek J. (2012). Expression of a disintegrin and metalloprotease in human abdominal aortic aneurysms. J. Vasc. Res..

[184] Vats S., Sundquist K., Wang X., Zarrouk M., Ågren-Witteschus S., Sundquist J., Gottsäter A., Memon A.A. (2020). Associations of global DNA methylation and homocysteine levels with abdominal aortic aneurysm: A cohort study from a population-based screening program in Sweden. Int. J. Cardiol..

[185] Toghill B.J., Saratzis A., Freeman P.J., Sylvius N., Bown M.J. (2018). SMYD2 promoter DNA methylation is associated with abdominal aortic aneurysm (AAA) and SMYD2 expression in vascular smooth muscle cells. Clin. Epigenet..

[186] Alexander M.R., Owens G.K. (2012). Epigenetic control of smooth muscle cell differentiation and phenotypic switching in vascular development and disease. Annu. Rev. Physiol..

[187] Hoshikawa Y., Matsuda Y., Suzuki S., Okada Y., Tabata T., Matsumura Y., Kondo T. (2005). Osteopontin may be responsible for pulmonary vascular remodeling. Chest.

[188] Lesauskaite V., Epistolato M.C., Castagnini M., Urbonavicius S., Tanganelli P. (2006). Expression of matrix metalloproteinases, their tissue inhibitors, and osteopontin in the wall of thoracic and abdominal aortas with dilatative pathology. Hum. Pathol..

[189] Zhang Y., Sun Z., Jia J., Du T., Zhang N., Tang Y., Fang Y., Fang D. (2021). Overview of histone modification. Histone Mutat. Cancer.

[190] Rodríguez-Castañeda F., Lemma R.B., Cuervo I., Bengtsen M., Moen L.M., Ledsaak M., Eskeland R., Gabrielsen O.S. (2018). The SUMO protease SENP1 and the chromatin remodeler CHD3 interact and jointly affect chromatin accessibility and gene expression. J. Biol. Chem..

[191] Han Y., Tanios F., Reeps C., Zhang J., Schwamborn K., Eckstein H.-H., Zernecke A., Pelisek J. (2016). Histone acetylation and histone acetyltransferases show significant alterations in human abdominal aortic aneurysm. Clin. Epigenet..

[192] Galán M., Varona S., Orriols M., Rodríguez J.A., Aguiló S., Dilmé J., Camacho M., Martínez-González J., Rodriguez C. (2016). Induction of histone deacetylases (HDACs) in human abdominal aortic aneurysm: Therapeutic potential of HDAC inhibitors. Dis. Models Mech..

[193] Vinh A., Gaspari T.A., Liu H.B., Dousha L.F., Widdop R.E., Dear A.E. (2008). A novel histone deacetylase inhibitor reduces abdominal aortic aneurysm formation in angiotensin II-infused apolipoprotein E-deficient mice. J. Vasc. Res..

[194] Xu Y., Zhang H., Chen Y., Pober J.S., Zhou M., Zhou J.H., Min W. (2024). SRF SUMOylation modulates smooth muscle phenotypic switch and vascular remodeling. Nat. Commun..

[195] Shi Y., Zhang H., Huang S., Yin L., Wang F., Luo P., Huang H. (2022). Epigenetic regulation in cardiovascular disease: Mechanisms and advances in clinical trials. Signal Transduct. Target. Ther..

[196] Liu H., Zhao Y., Zhao G., Deng Y., Chen Y.E., Zhang J. (2024). SWI/SNF complex in vascular smooth muscle cells and its implications in cardiovascular pathologies. Cells.

[197] Chang Z., Zhao G., Zhao Y., Lu H., Xiong W., Liang W., Sun J., Wang H., Zhu T., Rom O. (2020). BAF60a deficiency in vascular smooth muscle cells prevents abdominal aortic aneurysm by reducing inflammation and extracellular matrix degradation. Arterioscler. Thromb. Vasc. Biol..

[198] Zhao G., Zhao Y., Lu H., Chang Z., Liu H., Wang H., Liang W., Liu Y., Zhu T., Rom O. (2022). BAF60c prevents abdominal aortic aneurysm formation through epigenetic control of vascular smooth muscle cell homeostasis. J. Clin. Investig..

[199] Wang Y., Dong C.-Q., Peng G.-Y., Huang H.-y., Yu Y.-s., Ji Z.-C., Shen Z.-Y. (2019). MicroRNA-134-5p regulates media degeneration through inhibiting VSMC phenotypic switch and migration in thoracic aortic dissection. Mol. Ther. Nucleic Acids.

[200] Bi S., Peng Q., Liu W. (2020). MicroRNA-342-5p activates the Akt signaling pathway by downregulating PIK3R1 to modify the proliferation and differentiation of vascular smooth muscle cells. Exp. Ther. Med..

[201] Si X., Chen Q., Zhang J., Zhou W., Chen L., Chen J., Deng N., Li W., Liu D., Wang L. (2022). MicroRNA-23b prevents aortic aneurysm formation by inhibiting smooth muscle cell phenotypic switching via FoxO4 suppression. Life Sci..

[202] Sun L., Zhao M., Zhang J., Lv M., Li Y., Yang X., Liu A., Wu Z. (2017). MiR-29b downregulation induces phenotypic modulation of vascular smooth muscle cells: Implication for intracranial aneurysm formation and progression to rupture. Cell. Physiol. Biochem..

[203] Farina F.M., Hall I.F., Serio S., Zani S., Climent M., Salvarani N., Carullo P., Civilini E., Condorelli G., Elia L. (2020). miR-128-3p is a novel regulator of vascular smooth muscle cell phenotypic switch and vascular diseases. Circ. Res..

[204] Cluzel G.L., Ryan P.M., Herisson F.M., Caplice N.M. (2022). High-fidelity porcine models of metabolic syndrome: A contemporary synthesis. Am. J. Physiol.-Endocrinol. Metab..

[205] Tao W., Hong Y., He H., Han Q., Mao M., Hu B., Zhang H., Huang X., You W., Liang X. (2021). *MicroRNA*-199a-5p aggravates angiotensin II–induced vascular smooth muscle cell senescence by targeting Sirtuin-1 in abdominal aortic aneurysm. J. Cell. Mol. Med..

[206] Zhou J., Li Y.-S., Nguyen P., Wang K.-C., Weiss A., Kuo Y.-C., Chiu J.-J., Shyy J.Y., Chien S. (2013). Regulation of vascular smooth muscle cell turnover by endothelial cell–secreted microRNA-126: Role of shear stress. Circ. Res..

[207] Maegdefessel L., Spin J.M., Raaz U., Eken S.M., Toh R., Azuma J., Adam M., Nagakami F., Heymann H.M., Chernugobova E. (2014). miR-24 limits aortic vascular inflammation and murine abdominal aneurysm development. Nat. Commun..

[208] Yu X., Xu J.-f., Song M., Zhang L., Li Y.-h., Han L., Tang M.-x., Zhang W., Zhong M., Wang Z.-h. (2022). Associations of circulating microRNA-221 and 222 with the severity of coronary artery lesions in acute coronary syndrome patients. Angiology.

[209] Luo Y., Xiong W., Dong S., Liu F., Liu H., Li J. (2017). MicroRNA-146a promotes the proliferation of rat vascular smooth muscle cells by downregulating p53 signaling. Mol. Med. Rep..

[210] Zhao L., Ouyang Y., Bai Y., Gong J., Liao H. (2019). miR-155-5p inhibits the viability of vascular smooth muscle cell via targeting FOS and ZIC3 to promote aneurysm formation. Eur. J. Pharmacol..

[211] Lin W., Hou L., Tang J., Huang A., Jia Z. (2024). Mir-195-5p targets Smad7 regulation of the Wnt/β-catenin pathway to promote osteogenic differentiation of vascular smooth muscle cells. BMC Cardiovasc. Disord..

[212] Yang X., Dong M., Wen H., Liu X., Zhang M., Ma L., Zhang C., Luan X., Lu H., Zhang Y. (2017). MiR-26a contributes to the PDGF-BB-induced phenotypic switch of vascular smooth muscle cells by suppressing Smad1. Oncotarget.

[213] Jiang F., Zhang B., Zhang X., Zhang R., Lu Q., Shi F., Xu J., Deng L. (2023). miRNA-92a inhibits vascular smooth muscle cell phenotypic modulation and may help prevent in-stent restenosis. Mol. Med. Rep..

[214] Liu Y. (2019). Atherosclerotic Conditions Promote the Packaging of Functional microRNA-92 into Endothelial Microvesicles. Ph.D. Thesis.

[215] Hall I.F., Climent M., Quintavalle M., Farina F.M., Schorn T., Zani S., Carullo P., Kunderfranco P., Civilini E., Condorelli G. (2019). Circ_Lrp6, a circular RNA enriched in vascular smooth muscle cells, acts as a sponge regulating miRNA-145 function. Circ. Res..

[216] Rong Z.-H., Chang N.-B., Yao Q.-P., Li T., Zhu X.-L., Cao Y., Jiang M.-J., Cheng Y.-S., Jiang R., Jiang J. (2019). Suppression of circDcbld1 alleviates intimal hyperplasia in rat carotid artery by targeting miR-145-3p/Neuropilin-1. Mol. Ther. Nucleic Acids.

[217] Zeng Z., Xia L., Fan S., Zheng J., Qin J., Fan X., Liu Y., Tao J., Liu Y., Li K. (2021). Circular RNA CircMAP3K5 acts as a MicroRNA-22-3p sponge to promote resolution of intimal hyperplasia via TET2-mediated smooth muscle cell differentiation. Circulation.

[218] Thompson R.W., Liao S., Curci J.A. (1997). Vascular smooth muscle cell apoptosis in abdominal aortic aneurysms. Coron. Artery Dis..

[219] Lopez-Candales A., Holmes D.R., Liao S., Scott M.J., Wickline S.A., Thompson R.W. (1997). Decreased vascular smooth muscle cell density in medial degeneration of human abdominal aortic aneurysms. Am. J. Pathol..

[220] Huang C.-l., Huang Y.-n., Yao L., Li J.-p., Zhang Z.-h., Huang Z.-q., Chen S.-x., Zhang Y.-l., Wang J.-f., Chen Y.-x. (2023). Thoracic perivascular adipose tissue inhibits VSMC apoptosis and aortic aneurysm formation in mice via the secretome of browning adipocytes. Acta Pharmacol. Sin..

[221] Wang J., Da X., Chen Y., Yuan A., Pu J. (2024). Glutamine Protects against Mouse Abdominal Aortic Aneurysm through Modulating VSMC Apoptosis and M1 Macrophage Activation. Int. J. Med. Sci..

[222] Qu Y., Zhang N., Zhao Y. (2023). Resveratrol Inhibits Abdominal Aortic Aneurysm Progression by Reducing Extracellular Matrix Degradation, Apoptosis, Autophagy, and Inflammation of Vascular Smooth Muscle Cells via Upregulation of HMOX1. J. Endovasc. Ther..

[223] Wen Y., Liu Y., Li Q., Tan J., Fu X., Liang Y., Tuo Y., Liu L., Zhou X., LiuFu D. (2024). Spatiotemporal ATF3 Expression Determines VSMC Fate in Abdominal Aortic Aneurysm. Circ. Res..

[224] Liang K., Cui M., Fu X., Ma J., Zhang K., Zhang D., Zhai S. (2021). LncRNA Xist induces arterial smooth muscle cell apoptosis in thoracic aortic aneurysm through miR-29b-3p/Eln pathway. Biomed. Pharmacother..

[225] Zhang D., Lu D., Xu R., Zhai S., Zhang K. (2022). Inhibition of XIST attenuates abdominal aortic aneurysm in mice by regulating apoptosis of vascular smooth muscle cells through miR-762/MAP2K4 axis. Microvasc. Res..

[226] Ouyang Y., Hong Y., Mai C., Yang H., Wu Z., Gao X., Zeng W., Deng X., Liu B., Zhang Y. (2024). Transcriptome analysis reveals therapeutic potential of NAMPT in protecting against abdominal aortic aneurysm in human and mouse. Bioact. Mater..

[227] Lu H., Sun J., Liang W., Chang Z., Rom O., Zhao Y., Zhao G., Xiong W., Wang H., Zhu T. (2020). Cyclodextrin prevents abdominal aortic aneurysm via activation of vascular smooth muscle cell transcription factor EB. Circulation.

[228] Li K., Wei M., Zhang D., Zhai S., Liu H. (2024). PANoptosis in vascular smooth muscle cells regulated by TNF-α/IL-1β can be a new target for alleviating the progression of abdominal aortic aneurysm. Physiol. Genom..

[229] Martinez-Pinna R., Lindholt J.S., Madrigal-Matute J., Blanco-Colio L.M., Esteban-Salan M., Torres-Fonseca M.M., Lefebvre T., Delbosc S., Laustsen J., Driss F. (2014). From tissue iron retention to low systemic haemoglobin levels, new pathophysiological biomarkers of human abdominal aortic aneurysm. Thromb. Haemost..

[230] Liao F., Wang L., Wu Z., Luo G., Qian Y., He X., Ding S., Pu J. (2023). Disulfiram protects against abdominal aortic aneurysm by ameliorating vascular smooth muscle cells pyroptosis. Cardiovasc. Drugs Ther..

[231] Cai H., Li H., Xiao X., Wang S., Liu R., Qin Y., Zhou Y., Yao C. (2025). TRAF6 promotes abdominal aortic aneurysm development by activating macrophage pyroptosis via the NLRP3/Caspase1/GSDMD pathway. FASEB J..

[232] Ye B., Fan X., Fang Z., Mao C., Lin L., Wu J., Zheng W., Cai X., Huang W., Lv Y. (2024). Macrophage-derived GSDMD promotes abdominal aortic aneurysm and aortic smooth muscle cells pyroptosis. Int. Immunopharmacol..

[233] Li M., Yang Y., Zong J., Wang Z., Jiang S., Fu X., He X., Li X., Xue Q., Wang J.-X. (2022). miR-564: A potential regulator of vascular smooth muscle cells and therapeutic target for aortic dissection. J. Mol. Cell. Cardiol..

[234] Sun L., Li X., Luo Z., Li M., Liu H., Zhu Z., Wang J., Lu P., Wang L., Yang C. (2023). Purinergic receptor P2X7 contributes to abdominal aortic aneurysm development via modulating macrophage pyroptosis and inflammation. Transl. Res..

[235] Yin Z., Zhang J., Zhao M., Liu J., Xu Y., Peng S., Pan W., Wei C., Zheng Z., Liu S. (2024). EDIL3/Del-1 prevents aortic dissection through enhancing internalization and degradation of apoptotic vascular smooth muscle cells. Autophagy.

[236] Zhang F., Li K., Zhang W., Zhao Z., Chang F., Du J., Zhang X., Bao K., Zhang C., Shi L. (2024). Ganglioside GM3 protects against abdominal aortic aneurysm by suppressing ferroptosis. Circulation.

[237] Zou H.-X., Qiu B.-Q., Lai S.-Q., Huang H., Zhou X.-L., Gong C.-W., Wang L.-J., Yuan M.-M., He A.-D., Liu J.-C. (2021). Role of ferroptosis-related genes in Stanford type a aortic dissection and identification of key genes: New insights from bioinformatic analysis. Bioengineered.

[238] He X., Xiong Y., Liu Y., Li Y., Zhou H., Wu K. (2024). Ferrostatin-1 inhibits ferroptosis of vascular smooth muscle cells and alleviates abdominal aortic aneurysm formation through activating the SLC7A11/GPX4 axis. FASEB J..

[239] Krebs J.R., Bellotti P., Valisno J.A.C., Su G., Sharma S., Kollareth D.J.M., Hartman J.B., Adithan A., Spinosa M., Kamat M. (2024). Pharmacologic Inhibition of Ferroptosis Attenuates Experimental Abdominal Aortic Aneurysm Formation. bioRxiv.

[240] Zhou Y., Chen Y., Cui Y., Gan N., Xiang Q., Li M., Zeng W., Zheng X.-L., Dai X., Peng J. (2025). Inhibition of VSMC Ferroptosis Mitigates Pathological Vascular Remodeling: A Novel Therapeutic Strategy for Abdominal Aortic Aneurysm. J. Cardiovasc. Transl. Res..

[241] Hu X., Hu L., Si X., Feng Q., Ma Y., Liu Z., He X., Shi B. (2025). Comprehensive Bioinformatics Analysis Reveals the Role of Shared Cuproptosis-and Ferroptosis-Related DEG DLD in Abdominal Aortic Aneurysm. J. Cell. Mol. Med..

[242] Mutailipu M., Zhang M., Ding W., Fan Y., Ye Y., Lu Z. (2023). Identification of cuproptosis-related biomarkers in aortic dissection: New insights from bioinformatic analysis. Res. Sq..

[243] Xiao X., Deng Z., Huang Z., Liang C., Chen Z., Xiao X., Liu D. (2025). Comprehensive analysis the role of cuproptosis related genes in abdominal aortic aneurysm. Ann. Med. Surg..

[244] Van den Bergh G., Opdebeeck B., D’Haese P.C., Verhulst A. (2019). The vicious cycle of arterial stiffness and arterial media calcification. Trends Mol. Med..

[245] Vine N., Powell J.T. (1991). Metalloproteinases in degenerative aortic disease. Clin. Sci..

[246] Newman K.M., Ogata Y., Malon A.M., Irizarry E., Gandhi R.H., Nagase H., Tilson M.D. (1994). Identification of matrix metalloproteinases 3 (stromelysin-1) and 9 (gelatinase B) in abdominal aortic aneurysm. Arterioscler. Thromb. J. Vasc. Biol..

[247] Nishimura K., Ohgi S., Nanba E. (2001). Expression of MMP-2, MMP-9 and TIMP-1 in the Wall of Abdominal Aortic Aneurysms. Yonago Acta Medica.

[248] Stepien K.L., Bajdak-Rusinek K., Fus-Kujawa A., Kuczmik W., Gawron K. (2022). Role of extracellular matrix and inflammation in abdominal aortic aneurysm. Int. J. Mol. Sci..

[249] Davis V., Persidskaia R., Baca-Regen L., Itoh Y., Nagase H., Persidsky Y., Ghorpade A., Baxter B.T. (1998). Matrix metalloproteinase-2 production and its binding to the matrix are increased in abdominal aortic aneurysms. Arterioscler. Thromb. Vasc. Biol..

[250] Miyagawa K., Ogata T., Ueyama T., Kasahara T., Nakanishi N., Naito D., Taniguchi T., Hamaoka T., Maruyama N., Nishi M. (2017). Loss of MURC/Cavin-4 induces JNK and MMP-9 activity enhancement in vascular smooth muscle cells and exacerbates abdominal aortic aneurysm. Biochem. Biophys. Res. Commun..

[251] Ramella M., Boccafoschi F., Bellofatto K., Follenzi A., Fusaro L., Boldorini R., Casella F., Porta C., Settembrini P., Cannas M. (2017). Endothelial MMP-9 drives the inflammatory response in abdominal aortic aneurysm (AAA). Am. J. Transl. Res..

[252] Wang X., Wang M., Zhou Z., Zou X., Song G., Zhang Q., Zhou H. (2023). SMOC2 promoted vascular smooth muscle cell proliferation, migration, and extracellular matrix degradation by activating BMP/TGF-β1 signaling pathway. J. Clin. Biochem. Nutr..

[253] Longo G.M., Buda S.J., Fiotta N., Xiong W., Griener T., Shapiro S., Baxter B.T. (2005). MMP-12 has a role in abdominal aortic aneurysms in mice. Surgery.

[254] Koch A.E., Kunkel S.L., Pearce W.H., Shah M.R., Parikh D., Evanoff H.L., Haines G.K., Burdick M.D., Strieter R.M. (1993). Enhanced production of the chemotactic cytokines interleukin-8 and monocyte chemoattractant protein-1 in human abdominal aortic aneurysms. Am. J. Pathol..

[255] Papalambros E., Sigala F., Georgopoulos S., Menekakos C., Giatromanolaki A., Bastounis E., Sivridis E. (2003). Immunohistochemical expression of metalloproteinases MMP-2 and MMP-9 in abdominal aortic aneurysms: Correlation with symptoms and aortic diameter. Int. J. Mol. Med..

[256] Xie Z., Fang T. (2020). The expression and significance of NF-ÎºB, MMP1, and MMP2 in rats with abdominal aortic aneurysm. Cell. Mol. Biol..

[257] Curci J.A., Liao S., Huffman M.D., Shapiro S.D., Thompson R.W. (1998). Expression and localization of macrophage elastase (matrix metalloproteinase-12) in abdominal aortic aneurysms. J. Clin. Investig..

[258] Mao D., Lee J.K., VanVickle S.J., Thompson R.W. (1999). Expression of collagenase-3 (MMP-13) in human abdominal aortic aneurysms and vascular smooth muscle cells in culture. Biochem. Biophys. Res. Commun..

[259] Wilson W.R.W., Anderton M., Schwalbe E.C., Jones J.L., Furness P.N., Bell P.R., Thompson M.M. (2006). Matrix metalloproteinase-8 and-9 are increased at the site of abdominal aortic aneurysm rupture. Circulation.

[260] Brophy C.M., Marks W.H., Reilly J.M., Tilson M.D. (1991). Decreased tissue inhibitor of metalloproteinases (TIMP) in abdominal aortic aneurysm tissue: A preliminary report. J. Surg. Res..

[261] Elmore J.R., Keister B.F., Franklin D.P., Youkey J.R., Carey D.J. (1998). Expression of matrix metalloproteinases and TIMPs in human abdominal aortic aneurysms. Ann. Vasc. Surg..

[262] Eskandari M.K., Vijungco J.D., Flores A., Borensztajn J., Shively V., Pearce W.H. (2005). Enhanced abdominal aortic aneurysm in TIMP-1-deficient mice1. J. Surg. Res..

[263] Hu M., Meganathan I., Zhu J., MacArthur R., Kassiri Z. (2023). Loss of TIMP3, but not TIMP4, exacerbates thoracic and abdominal aortic aneurysm. J. Mol. Cell. Cardiol..

[264] Zhong S., Khalil R.A. (2019). A Disintegrin and Metalloproteinase (ADAM) and ADAM with thrombospondin motifs (ADAMTS) family in vascular biology and disease. Biochem. Pharmacol..

[265] Zhang P., Shen M., Fernandez-Patron C., Kassiri Z. (2016). ADAMs family and relatives in cardiovascular physiology and pathology. J. Mol. Cell. Cardiol..

[266] Giebeler N., Zigrino P. (2016). A disintegrin and metalloprotease (ADAM): Historical overview of their functions. Toxins.

[267] Mizoguchi T., MacDonald B.T., Bhandary B., Popp N.R., Laprise D., Arduini A., Lai D., Zhu Q.M., Xing Y., Kaushik V.K. (2021). Coronary disease association with ADAMTS7 is due to protease activity. Circ. Res..

[268] Geng L., Wang W., Chen Y., Cao J., Lu L., Chen Q., He R., Shen W. (2010). Elevation of ADAM10, ADAM17, MMP-2 and MMP-9 expression with media degeneration features CaCl2-induced thoracic aortic aneurysm in a rat model. Exp. Mol. Pathol..

[269] Jana S., Chute M., Hu M., Winkelaar G., Owen C.A., Oudit G.Y., Kassiri Z. (2020). ADAM (a disintegrin and metalloproteinase) 15 deficiency exacerbates Ang II (angiotensin II)–Induced aortic remodeling leading to abdominal aortic aneurysm. Arterioscler. Thromb. Vasc. Biol..

[270] Fava M., Barallobre-Barreiro J., Mayr U., Lu R., Didangelos A., Baig F., Lynch M., Catibog N., Joshi A., Barwari T. (2018). Role of ADAMTS-5 in aortic dilatation and extracellular matrix remodeling. Arterioscler. Thromb. Vasc. Biol..

[271] Qiu R., Chen S., Gao P., Luo K., Feng X., Yuan H., Wu X., Li G. (2021). ADAM10 attenuates the development of abdominal aortic aneurysms in a mouse model. Mol. Med. Rep..

[272] Jiao T., Yao Y., Zhang B., Hao D.-C., Sun Q.-F., Li J.-B., Yuan C., Jing B., Wang Y.-P., Wang H.-Y. (2017). Role of MicroRNA-103a targeting ADAM10 in abdominal aortic aneurysm. BioMed Res. Int..

[273] Shen G., Sun Q., Yao Y., Li S., Liu G., Yuan C., Li H., Xu Y., Wang H. (2020). Role of ADAM9 and miR-126 in the development of abdominal aortic aneurysm. Atherosclerosis.

[274] Oller J., Méndez-Barbero N., Ruiz E.J., Villahoz S., Renard M., Canelas L.I., Briones A.M., Alberca R., Lozano-Vidal N., Hurlé M.A. (2017). Nitric oxide mediates aortic disease in mice deficient in the metalloprotease Adamts1 and in a mouse model of Marfan syndrome. Nat. Med..

[275] Wang S., Liu Y., Zhao G., He L., Fu Y., Yu C., Wang Z., Zhao T., Cao F., Gao Y. (2018). Postnatal deficiency of ADAMTS1 ameliorates thoracic aortic aneurysm and dissection in mice. Exp. Physiol..

[276] Fashandi A.Z., Spinosa M., Salmon M., Su G., Montgomery W., Mast A., Lu G., Hawkins R.B., Cullen J.M., Sharma A.K. (2020). Female mice exhibit abdominal aortic aneurysm protection in an established rupture model. J. Surg. Res..

[277] Ghosh A., Lu G., Su G., McEvoy B., Sadiq O., DiMusto P.D., Laser A., Futchko J.S., Henke P.K., Eliason J.L. (2014). Phosphorylation of AKT and abdominal aortic aneurysm formation. Am. J. Pathol..

